# Radiation synthesis of sodium alginate/gelatin based ultra-absorbent hydrogel for efficient water and nitrogen management in wheat under drought stress

**DOI:** 10.1038/s41598-024-69333-3

**Published:** 2024-08-22

**Authors:** Mahmoud A. El-diehy, Ibrahim I. Farghal, Mohamed A. Amin, Mohamed mohamady Ghobashy, Abdelatti I. Nowwar, H. M. Gayed

**Affiliations:** 1 Botany and Microbiology Department, Faculty of Science, Al-Azhar University, Cairo, Egypt; 2 Radiation Research of Polymer Chemistry Department, National Center for Radiation Research and Technology (NCRRT), Egyptian Atomic Energy Authority (EAEA), Cairo, Egypt

**Keywords:** Ultra-absorbent, Biopolymer, Nitrogen management, Radiation, Climate sciences, Environmental sciences, Chemistry

## Abstract

The main focus of this study was on using radiation to make an ultra-absorbent hydrogel (UAH) from sodium alginate (SA) and gelatin (GL) biopolymers. This UAH can effectively handle water and nitrogen in wheat farming during drought stress. The hydrogel was synthesized by gamma irradiation-induced SA/GL/polyacrylamide crosslinking at 10–40 kGy. Varying SA/GL ratios affected swelling and the gel fraction of SA/GL/PAm hydrogels. The (SA/GL 17/83) hydrogel exhibited a 40.03 g/g swelling degree, while increasing SA content resulted in higher swelling, peaking at 75.5 g/g for (SA/GL 83/17). This indicated a synergistic interaction between SA and GL. The gel fraction also increased from 76.8 to 90.3%, with a higher GL content reflecting increased crosslinking. After multiple hydrolysis cycles, the hydrogel achieved 1293 (g/g) swelling and 36 days of water retention. When applied to wheat (*Triticuma estivum*) under drought stress, it significantly improved shoot length (18%), root length (43%), shoot fresh weight (49%), and shoot dry weight (51%) under extreme drought. The significant increases in protein and carbohydrate content in both shoots (up to 32% and 19%, respectively) and grains (up to 21% and 24%, respectively), along with the reduction in proline content (up to 38%), demonstrate that ultra-absorbent hydrogel (UAH) effectively enhances nitrogen content, photosynthesis, and overall plant health in wheat under varying drought stress levels. This novel SA/GL-based UAH holds promise for addressing water scarcity and agricultural challenges, offering a sustainable solution for water and nitrogen management under drought stress.

## Introduction

Food security lies at the core of Egypt’s 2030 Vision, which is regarded as national security. Egypt’s wheat supply depends mainly on two sources: domestic wheat production and imported wheat. Egypt produces about half of the twenty million tons of wheat consumed through irrigated agriculture, and the other half is imported^1^. Wheat cultivation occupies about 33% of Egypt’s total winter crop, making it significant. It is mainly eaten as bread^2^. Wheat contributes more than one-third of the daily caloric intake of Egyptians and accounts for 45% of their total daily protein intake^2^. Despite a four-fold increase in total production over the past four decades, new growth opportunities have been limited by increasing water scarcity and agriculture due to the inhibition of expansion and gain^3^. Also, as climate change continues to alter traditional precipitation patterns, the importance of efficient water management in agriculture cannot be overstated^4^. Ultra-absorbent hydrogels (UAHs) have emerged as a promising tool for retaining and gradually releasing water in arid and water-scarce regions, improving crop resilience^5–7^. UAHs, particularly in agricultural applications for water preservation and soil conditioning^8,9^, have garnered significant attention as a solution to the ever-increasing challenges posed by climate change and the need for effective nitrogen management in the agricultural sector^10,11^.

In agricultural innovation, biopolymers such as sodium alginate and gelatin herald a groundbreaking approach to engineering hydrogels with properties meticulously designed for water management in diverse agricultural settings^8,9^. Sodium alginate provides water retention to hydrogels, while gelatin adds biodegradability, enhancing nutrient release^12^*.* Sodium alginate, derived from seaweed, and gelatin, a protein-based biopolymer, exhibit unique properties that, when combined, lead to a hydrogel with exceptional versatility^13^. The intricate interplay between these biopolymers results in a hydrogel matrix with enhanced structural integrity and resilience, well-suited for various biomedical and agricultural applications^14,15^. The specific hydrogel characteristics crucial for agricultural water management, such as water retention, nutrient availability, and drought resilience, are tailored through a meticulous engineering process^13,16^. Biopolymers, derived from renewable sources, play a pivotal role in sustainable agriculture^17^ and packaging^18^, helping reduce the environmental footprint while ensuring the efficient use of resources^19^. Effective nitrogen management is equally vital, as it strikes a balance between increasing crop yields and minimizing nitrogen-related ecological issues, thus reinforcing the sustainability of agriculture^20^. Moreover, using gamma irradiation as a hydrogel synthesis method offers several benefits, including precise control over the hydrogel's properties, cross-linking efficiency, and sterility^21–26^. This introduction sets the stage for a comprehensive exploration of these interconnected topics and their profound effects on the agricultural sector, where innovative hydrogel solutions promise sustainable and climate-resilient farming practices^27^.

The novelty of this study lies in the innovative utilization of biopolymers, specifically sodium alginate (SA) and gelatin (GL), within the hydrogel formulation to rationalize the water consumption of wheat plants. Biopolymers such as SA and GL are derived from natural sources, making them eco-friendly and sustainable alternatives to synthetic polymers. Combining these biopolymers in the (SA/GL/PAm) hydrogel showcases their unique ability to influence structural integrity and water-absorbing properties. The synergy between SA and GL allows for precisely controlling hydrogel characteristics, opening doors to biomedicine, agriculture, and environmental science applications. The novelty here is in harnessing the potential of biopolymers to engineer hydrogels with tailored properties, addressing a wide range of real-world challenges. This study proposes that using sustainable materials, such as sodium alginate and gelatin, in developing an ultra-absorbent hydrogel will provide an effective solution to enhance water management in agriculture. The hypothesis posits that these biodegradable hydrogels can significantly improve soil water retention, reduce water consumption, and promote sustainable crop cultivation.

## Experimental

### Materials

SA (chemical grade, 99%, Mw = 30 kDa, M/G ratio = 0.63) and GL (buffalo skin) were supplied from Sigma–Aldrich Co. and used as received. Acrylamide monomer (99%) AAm was supplied from Fluka Co. Potassium hydroxide was purchased from the Aladdin Industrial Corporation (Shanghai, China). The Fertilizer (urea) were purchased from El-Gomhorea company in Egypt. Seeds of *Triticuma estivum (*wheat plants) Var. Sakkha 95 was supplied from Sakkha Research Center, Agriculture Ministry, Kafr el-Sheikh, Egypt. The present study complies with relevant institutional, national, and international guidelines and legislation. The researchers performed field studies using commercially available plant varieties. No appropriate permissions and/or licences for the collection of plant or seed specimens were required to conduct this research.

### Radiation synthesis of (SA/GL/PAm) hydrogel

**Figure 1:**
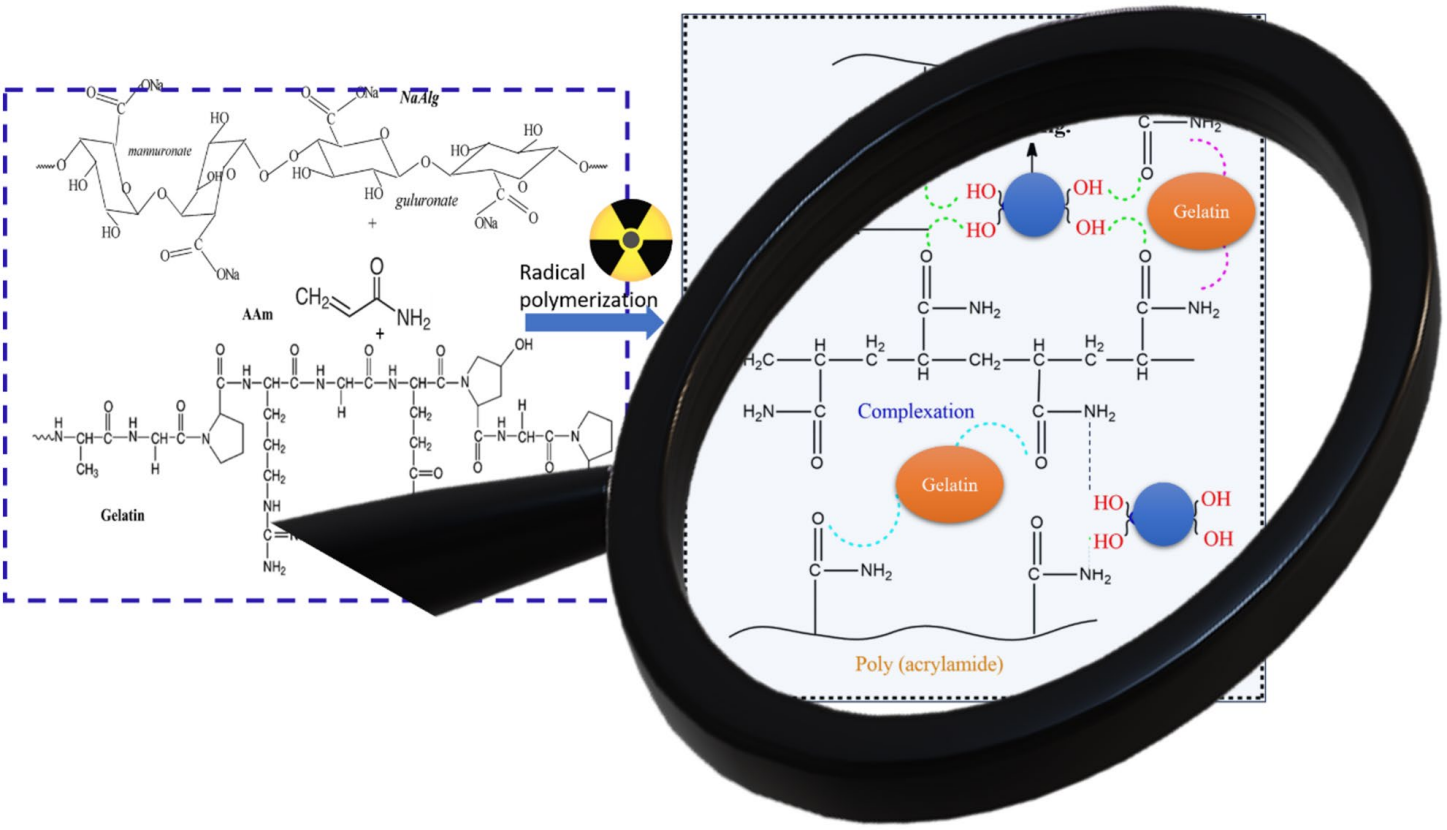
Schematic representation of (SA/GL/PAm) hydrogel formation by gamma ray irradiation.

The preparation of hydrogels using a combination of natural and synthetic polymers through radiation-induced cross-linking. It’s important to note that the choice of radiation dose and the concentration of the components can be adjusted to tailor the properties of the resulting hydrogel to specific applications. Start by preparing a solution of different concentrations of SA and GL dissolved in distilled water. Aqueous solutions of SA (1%) and GL (1%) were prepared in different volume ratios: 17/83, 34/66, 50/50, 66/34, and 83/17. These two components serve as the base for the hydrogel. After preparing the SA/GL solution, different concentrations of acrylamide monomer (2.5, 5, 10, and 15%) were added to this solution. Acrylamide will become integral to the hydrogel and cross-link with the SA and GL during irradiation. Thoroughly mix the SA/GL/acrylamide solution for 3 h to ensure the components are well-mixed and homogeneous. This is important to achieve consistent properties in the resulting hydrogel. Once the mixture is ready, the next step is to expose it to gamma rays. Gamma irradiation-induced free radical cross-linking copolymerization is used to form the hydrogel. The irradiation is done at different doses (10, 20, 30, and 40 kGy) using a Co60 γ-cell-220 source. The radical polymerization occurs within the acrylamide network, and hydrogen bonds are established between acrylamide and gelatin as well as between acrylamide and sodium alginate as shown in Fig. 1. The Co60 γ-cell-220 source was established at the National Center for Radiation Research and Technology (NCRRT), Egyptian Atomic Energy Authority (EAEA) in Cairo, Egypt. The irradiation process is conducted at a dose rate of approximately 0.83 kGy/h. After irradiation, the hydrogel samples are cut into discs and washed in deionized water for 24 h. This washing step helps remove any unreacted monomers or impurities from the hydrogel. The hydrogels are then oven-dried to remove excess water. This step is crucial for preserving the structure and properties of the hydrogel. Finally, the dried hydrogels are cut into small pieces for further analysis. The analysis may include characterizing the hydrogel’s physical and chemical properties.

### Chemical modification of (SA/GL/PAm) hydrogel to ultra-absorbent hydrogel (UAH)

The specific conditions and parameters used for this hydrolysis process, as described in the previous work^7^, are critical in achieving this desired level of swelling and water absorbance. The choice of alkali (in this case, KOH), concentration, temperature, and reaction time all play a role in determining the final properties of the UAH. The hydrolysis of (SA/GL/PAm) was carried out by reaction with 10 wt% of KOH at a temperature of 90 °C for 90 min. The reaction rate is initially relatively high, and hydrolysis is rapid. This is attributed to the availability of many amide groups in the polymer chain that can readily react with the hydroxide ions. The hydrolysis of amide bond groups (–CONH_2_) by KOH is outlined as the following Eq. ^28,29^:
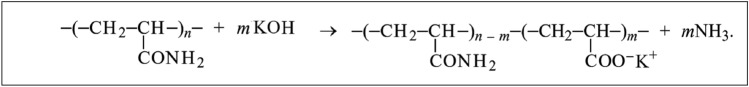


### Characterization

The chemical structure and surface morphology of the (SA/GL/PAm) hydrogels were analyzed using Fourier transform infrared spectrophotometry (FTIR) with an attenuated total reflectance (ATR) accessory on a Vertex 70 FTIR spectrometer equipped with a HYPERION^™^ series microscope from Bruker Optik GmbH, Ettlingen, Germany. FTIR allowed for the identification of functional groups and chemical bonds in the hydrogel. Additionally, surface morphology was examined using a ZEISS EVO 15 scanning electron microscope (SEM) located in the United Kingdom (UK), providing high-resolution images that revealed surface topography, structural features, and the size and distribution of pores. These combined analyses offered comprehensive insights into the chemical and physical characteristics of the hydrogel, supporting its evaluation for various applications.

### Analysis of the gel fraction (%) and swelling degree, water retention

To measure the gel fraction **(%)** of the obtained (SA/GL/PAm) hydrogel, the initial weight (*W*_*i*_) after the prepared step was measured. Then, the (SA/GL/PAm) hydrogel was immersed in 100 ml of distilled water and on standby for 48 h at ambient temperature. The distilled water was refreshed twice every five hours. The swollen (SA/GL/PAm) hydrogel piece was placed in an oven at a temperature of 50 °C until completely dry. The dried weight was measured (*W*_*d*_), and the gel fraction (%) was calculated using Eq. (1).

1 $${\text{Gel fraction }}\left( {\text{\% }} \right){ = }\left( {{\text{W}}_{{\text{d}}} {\text{/W}}_{{\text{i}}} } \right) \, \times { 100}$$

To calculate the swelling degree and equilibrium swelling, the dried weight of the obtained (SA/GL/PAm) hydrogel was measured (*W*_*d*_) and immersed in distilled water to measure the swelling rate with time (t) to give the swelled weight (*W*_*t*_).

2 $${\text{Swelling degree }}\left( {\text{g/g}} \right){ = }\left( {W_{t} - W_{d} } \right){/}W_{d}$$

After 48 h, the water absorption equilibrium of the (SA/GL/PAm) hydrogel was reached (*W*_*ES*_), and the swelling equilibrium was calculated as Eq. (3)

3 $${\text{Swelling equilibrium }}\left( {\text{g/g}} \right){ = }\left( {W_{t} - W_{o} } \right){/}\left( {W_{o} - W_{ES} } \right) \, \times {100}$$

The water retention (%) was calculated by putting the weighted (SA/GL/PAm) hydrogel at maximum swelling in equilibrium (*W*_*ES*_) the weight of hydrogel at a given time (*W*_*t*_), and the weight of the dried hydrogel (*W*_*o*_)

4 $${\text{The water retention }}\left( {\text{\% }} \right){ = }\left( {W_{t} - W_{o} } \right){/}\left( {W_{o} - W_{ES} } \right) \, \times {100}$$

The trial optimum condition was repeated three times to achieve high confidence and reproducibility.

### Evaluation of the effect of ultra-absorbent hydrogel (UAH) on the growth of wheat plants under drought stress

The study was done on sandy loam soil in El Gharbia, Egypt. The soil physiochemical analysis (analysis at Ain Shams University, Faculty of Agriculture, Cairo, Egypt, in the arid land research and services center) is displayed in Table 1s. The experiment involved the application of 5 g of UAH (loaded with 5% urea) to each seed of wheat plants. The supplementary file details the treatment process (Table 2s and Fig. 1s).

## Result and discussion

### FTIR analysis of (SA/GL/PAm) before and after hydrolysis

**Figure 2:**
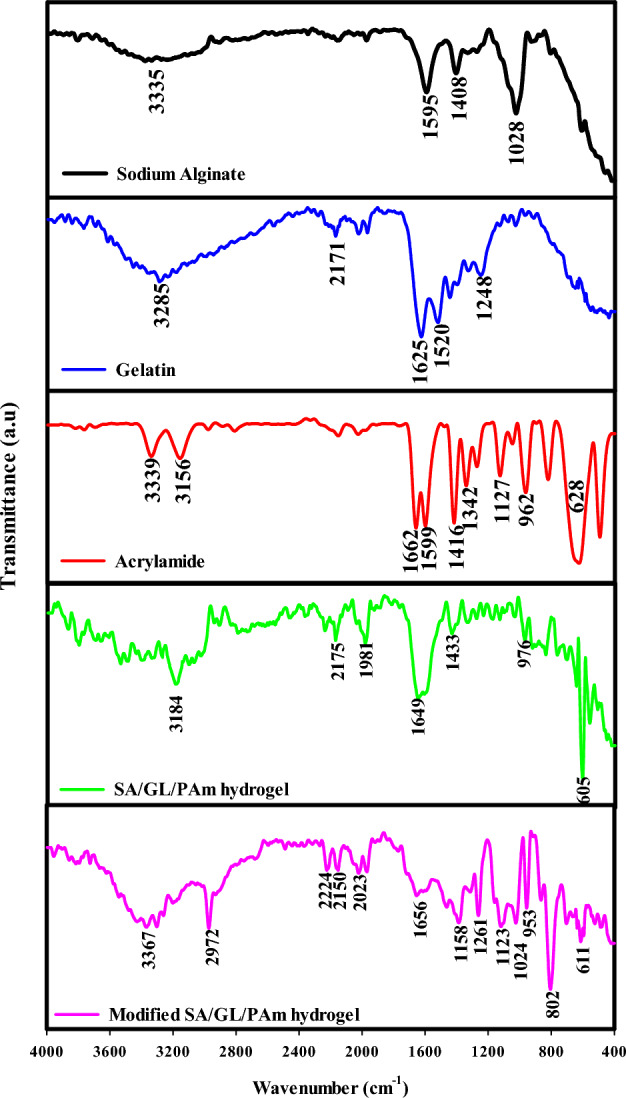
FTIR of sodium alginate, gelatin, acrylamide, SA/GL/PAm hydrogel, and modified SA/GL/PAm hydrogel.

In Fig. 2, the FTIR spectra revealed distinct features that shed light on these components' chemical identities and interactions. Sodium alginate, a natural polymer derived from brown algae, displayed a pronounced peak at 3335 cm^−1^, a signature of OH stretching vibrations. Moreover, the spectra exhibited distinct absorption bands at 1595 cm^−1^ and 1408 cm^−1^, which can be attributed to carboxylate groups' asymmetric and symmetric stretching vibrations^30^. This corroborates the presence of carboxylic groups within sodium alginate, an essential component in many hydrogel applications. Gelatin, another vital constituent in this hydrogel composite, presented a broad OH stretching peak at 3285 cm^−1^, indicating its abundant hydroxyl groups. Notably, gelatin is a hydrolyzed form of collagen with distinctive bands at 1625 cm^−1^, 1520 cm^−1^, and 1248 cm^−1^^31^. These bands correspond to amide I, amide II, and amide III vibrations, reflecting the proteinaceous nature of gelatin. The rich spectrum of gelatin testifies to its vital role in contributing to the hydrogel's mechanical stability and protein-based structure. The SA/GL/PAm hydrogel, made up of sodium alginate, gelatin, and acrylamide, showed a spectrum that showed the unique properties of each of its polymers. Some changes were seen in the spectra, mainly in the bands' wavenumbers corresponding to sodium alginate and gelatin. However, the peaks from each component made it clear that they worked together and were connected without covalent bonds within the hydrogel. Additionally, the absence of a sharp band at 1662 cm^−1^, indicative of the C = C bond of acrylamide, is compelling evidence of radiation-induced polymerization during the hydrogel’s synthesis.

Interestingly, the FTIR analysis of the modified SA/GL/PAm (UAH) showed the big changes that were taking place in this advanced material. The spectra exhibited a noticeable shift in the (–NH_2_) peak from 3184 cm^−1^ in the SA/GL/PAm hydrogel to 3367 cm^−1^ in the UAH. This change, along with a lower peak intensity and a wider peak, clearly showed that the amide bond groups in the modified hydrogel's structure were being broken down by water. This transformation is paramount, as it enhances the hydrogel’s water-absorbing capabilities, making it an efficient tool for water management, soil conditioning, and various agricultural applications.

### Effect of biopolymer (SA/GL) content on the gel fraction and swelling degree of SA/GL/PAm hydrogel

SA and GL are critical building blocks in the (SA/GL/PAm) hydrogel, impacting its gel fraction and water-absorbing properties. The concentration of biopolymers, specifically the ratio of SA to GL, plays a pivotal role in shaping the characteristics of the resulting hydrogel^32^. The gel fraction shows how much the polymer chains have crossed-linked to form a network structure. It is an important parameter that shows how structurally stable the hydrogel is and how well it can absorb water. The SA-to-GL ratio within the hydrogel formulation can be optimized to achieve specific properties and characteristics. The choice of ratio can be tailored to meet the requirements of a particular application. For instance, a hydrogel with a balanced SA and GL ratio may offer a compromise between water-absorbing capacity and gelation percentage, suitable for various applications.

**Figure 3:**
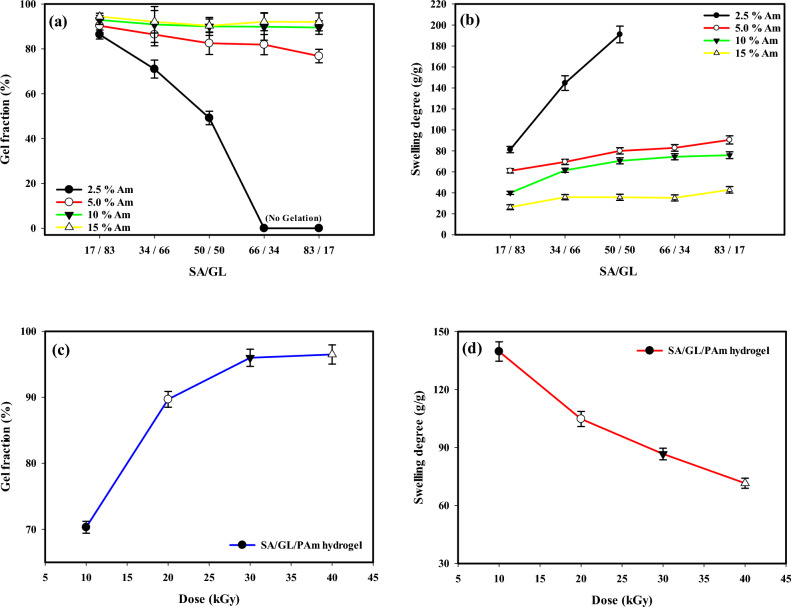
Effect of biopolymer (SA/GL) content on the gel fraction (**a**) and swelling degree (**b**) of SA/GL/PAm hydrogel, the effect of gamma radiation doses on the gel fraction (**c**) and swelling degree (**d**) of SA/GL/PAm hydrogel.

Figure 3a shows that the ratio of sodium alginate to gelatin in the hydrogel composition influences the gel fraction percentage. A higher proportion of gelatin tends to result in a higher gel fraction, while a higher proportion of sodium alginate reduces the gel fraction. The ratio choice can be tailored for various applications to achieve specific properties and characteristics in the resulting hydrogel. At a 17/83 SA/GL ratio, the acrylamide content is 2.5%, and the gel fraction percentage is 86.4%. This indicates a relatively low acrylamide content and a significant gel fraction. At a 34/66 and 50/50 SA/GL ratio, the gel fraction percentages are 71% and 49.2%, respectively. At 66/34 and 83/17 SA/GL ratios, the acrylamide content is 2.5% and the gel fraction percentage is 0%. In this case, the gel fraction is minimal, indicating a lack of gel formation as SA content increases^33^. Gel fraction also increased from 76.8 to 90.3% with higher GL content, reflecting increased crosslinking (at 5% acrylamide content). At (17/83, 34/66, 50/50, 66/34, and 83/17 SA/GL) hydrogel with acrylamide content 10% Am, the gel fraction percentages are 92.9%, 90.9%, 90%, 89.9%, and 89.5%, respectively. At a 66/34 SA/GL ratio, the acrylamide content is 15%, and the gel fraction percentage is 92.1%. The acrylamide content has significantly increased, and the gel fraction remains high.

Figure 3b reveals that the amount of biopolymer present, specifically the ratio of SA to GL, is a key factor in determining how the hydrogel swells. The swelling percentage, which quantifies the extent to which the hydrogel can absorb and retain water, is a crucial parameter reflecting the hydrogel’s performance in various applications. The SA/GL ratio can be carefully adjusted to achieve specific properties and characteristics tailored to the requirements of a particular application. For instance, a balanced SA and GL ratio may compromise water-absorbing capacity and gelation percentage, making it suitable for various applications. The pivotal role of biopolymers, namely SA and GL, in shaping the swelling behavior of (SA/GL/PAm) hydrogel underscores their significance in hydrogel engineering. These biopolymers contribute to the hydrogel's water-absorbing properties and influence its structural integrity and gelation percentage. The versatility of biopolymers allows for precise control over hydrogel properties, making them ideal candidates for a wide range of applications. Figure 3b reveals the profound influence of the SA/GL ratio on the properties of the hydrogel. At a 17/83 SA/GL ratio, the acrylamide content is 2.5%, and the swelling degree is 81.3 (g/g). Transitioning to a 34/66 SA/GL ratio maintains the acrylamide content at 2.5% but significantly enhances the swelling to 144.6 g/g. As the SA and GL components approach a balanced 50/50 ratio at an acrylamide content of 5%, the swelling degree remains respectable at 98 g/g. Moving towards a 66/34 SA/GL ratio, the acrylamide content is 5%, and the swelling degree improves to 82.85%. At 5% acrylamide content, the (SA/GL 17/83) hydrogel exhibited a 61 (g/g) swelling degree, while increasing SA content resulted in higher swelling, peaking at 91.5 (g/g) for (SA/GL 83/17). Finally, at 10% acrylamide content, the (SA/GL 17/83) hydrogel exhibited a 40.03 (g/g) swelling degree, while increasing SA content resulted in higher swelling, peaking at 75.5 (g/g) for (SA/GL 83/17). The findings show that the balance between SA and GL is crucial for controlling the hydrogel's ability to absorb water and its acrylamide content. This information can make the hydrogel’s properties fit specific uses where water absorption is important.

### Effect of gamma radiation doses on the gel fraction and swelling degree of SA/GL/PAm hydrogel

Figure 3c demonstrates the relationship between the irradiation dose (kGy) and the resulting gel fraction percentage of the (SA/GL/PAm) hydrogel. This relationship highlights the significant impact of irradiation on the gel fraction, an essential parameter reflecting the hydrogel’s structural integrity and water-absorbing capacity. At an irradiation dose of 10 kGy, the gel fraction percentage is 70.3%. This indicates that the hydrogel’s network structure has formed somewhat, but there is room for further improvement. A gel fraction of 70.3% suggests that the hydrogel may have a moderate water-absorbing capacity. At an irradiation dose of 20 kGy, the gel fraction percentage significantly increases to 89.7%. This higher irradiation dose produces a much denser network structure^34^, indicating enhanced structural integrity and water-absorbing capacity. The hydrogel is now better suited for applications requiring efficient water retention. The gel fraction percentage rises at an irradiation dose of 30 kGy, reaching 96%. This dose further optimizes the hydrogel’s network structure, maximizing its structural integrity and water-absorbing capacity.

A gel fraction of 96% signifies that the hydrogel is well-suited for applications demanding high water retention. At an irradiation dose of 40 kGy, the gel fraction percentage reaches 96.5%, indicating a minor increase in structural integrity compared to the 30 kGy dose. The network structure is nearly fully developed, and the hydrogel possesses exceptional water-absorbing capabilities. The slight increase in the gel fraction suggests that further irradiation may have diminishing returns regarding structural improvement. These results underscore the pivotal role of irradiation dose in shaping the structural characteristics and water-absorbing properties of (SA/GL/PAm) hydrogel. The data shows that a higher irradiation dose makes the network structure denser and better at retaining water. The 30 kGy dose best balances irradiation intensity and structural integrity. This information is invaluable for tailoring hydrogel properties to meet the requirements of specific applications, whether in agriculture, medicine, or environmental management. Figure 3d illustrates the impact of irradiation dose (measured in kGy) on the swelling percentage of the (SA/GL/PAm) hydrogel. These findings reveal a significant relationship between the irradiation dose and the hydrogel's water-absorbing capacity. At an irradiation dose of 10 kGy, the hydrogel exhibits a substantial swelling percentage of 139.7%, indicating its ability to efficiently absorb and retain water. As the irradiation dose is increased to 20 kGy, the swelling percentage decreases to 104.8%. This suggests a higher irradiation dose can reduce the hydrogel's water-absorbing capacity, indicating a denser network structure. Further increasing the irradiation dose to 30 kGy results in a decreased swelling percentage of 86.68%. This trend continues to emphasize the impact of irradiation on the hydrogel’s water-absorbing properties. Finally, when the irradiation dose is raised to 40 kGy, the swelling percentage drops even more to 71.5%. This shows that a higher irradiation dose makes the network structure denser and lowers its ability to absorb water^35^. These findings underscore the critical role of irradiation dose in shaping the swelling behavior of the (SA/GL/PAm) hydrogel. The data suggests that the irradiation dose can be carefully adjusted to achieve specific water-absorbing properties tailored to the requirements of different applications, particularly in fields where controlled water absorption is crucial.

### Effect of hydrolysis cycling number on the swelling degree and its kinetics

**Figure 4:**
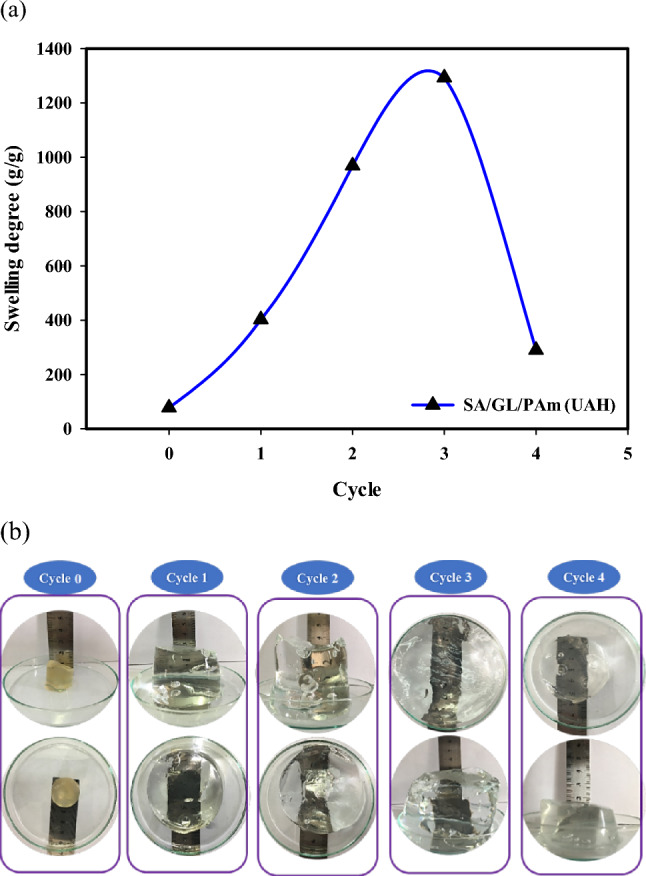
The equilibrium swelling degree (g/g) (**a**) and photograph (**b**) of the UAH at different modification cycles (0–4).

The hydrolysis of the (SA/GL/PAm) hydrogel is a critical process that can significantly impact its swelling behavior and water-absorbing capacity. In this part, we’ll look at how cycling hydrolysis affects the amount of swelling and how fast it happens. This will help us understand how the hydrogel changes when it's broken down in different ways. The hydrolysis process of the (SA/GL/PAm) hydrogel involves subjecting it to an aqueous potassium hydroxide (KOH) solution under controlled conditions, explicitly utilizing a 10 wt% KOH solution, maintaining a temperature of 90 °C, and allowing the reaction to proceed for 90 min. The aim is to examine how multiple hydrolysis cycles impact the hydrogel's water-absorbing capacity. The relationship between the hydrolysis cycles and the water-absorbing capacity of the hydrogel is presented in Fig. 4a. The data reveals a clear correlation between the number of cycles and the swelling percentage (g/g). As shown, absorbency increases with an increase in the hydrolysis cycle.

**Figure 5:**
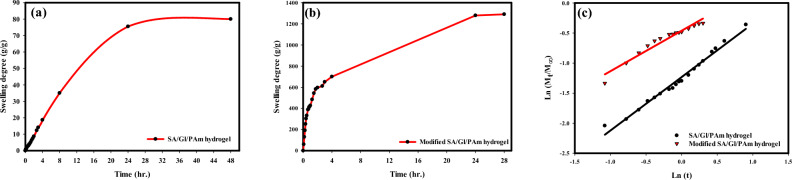
Rate of swelling (**a**,** b**) and swelling kinetics (**c**) of SA/GL/PAm hydrogel and UAH, respectively.

The initial swelling degree is recorded at 77.5 (g/g), and with the first hydrolysis cycle, it increases substantially to 402.3 (g/g). The swelling percentage rises in the second cycle, reaching 969 (g/g). However, the third cycle demonstrates the most remarkable increase, achieving a swelling degree of 1293 (g/g). In contrast, the fourth cycle experiences a reduction in swelling, dropping to 290 (g/g). This intriguing pattern suggests an optimal range of hydrolysis cycles where the hydrogel’s water-absorbing capacity is maximized. The corresponding photographs in Fig. 4b illustrate the hydrogel’s appearance after undergoing different hydrolysis processes. Despite its expansion, the hydrogel maintains its initial cylindrical form when swollen. The photographs also reflect the variations in size and swelling behavior with each cycle, with the third cycle displaying the most significant increase in height and diameter. Figure 5a, b show how the swelling rate changes for the SA/GL/PAm hydrogel and the UAH. It is evident that, with increasing time, the swelling rate experiences a steady increase until it reaches a point of stability, which occurs after approximately 20 h for both hydrogels. This suggests a specific equilibrium state is achieved, where the hydrogel's water-absorbing capacity remains relatively constant. The analysis of the diffusion mechanism for both hydrogels indicates that the swelling follows a non-Fickian diffusion mechanism (Fig. 5c). The non-Fickian diffusion mechanism is characterized by a combination of diffusion and relaxation processes, implying that the swelling behavior is not solely dependent on the concentration gradient but also on the relaxation of the polymer network. The SA/GL/PAm hydrogel and the UAH’s diffusion parameters are shown in Table 1, along with their n-values, which show that the diffusion mechanism is non-Fickian. These values, which are less than 1 and more than 0.5, further support the idea that the swelling is influenced by a combination of diffusion and relaxation processes rather than purely Fickian^36^.

**Table 1:** Diffusion parameters of the SA/GL/PAm hydrogel and UAH.

Hydrogel	n	Mechanism	R^2^
SA/GL/PAm	0.8893	Non-Fickian	0.9839
UAH	0.6729	Non-Fickian	0.9448

### Surface morphology analysis of SEM before and after hydrolysis

**Figure 6:**
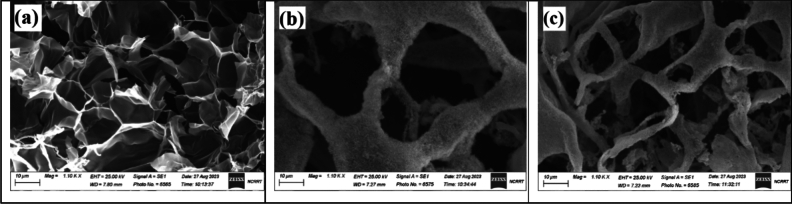
SEM of SA/GL/PAm hydrogel (**a**) and UAH after hydrolysis (**b**, **c**).

The cross-sectional shape of the swollen SA/GL/PAm hydrogel before and after chemical modification, as shown in Fig. 6, gives us interesting information about how this material is put together. The hydrogel exhibits a distinctive surface morphology characterized by three-dimensional pores (Fig. 6a). Notably, these pores appear relatively uniform, boasting a diameter of approximately 10 µm. This regularity in pore size contributes to the hydrogel’s consistent and efficient water-absorbing capacity. In contrast, the UAH displays notable deviations in morphology compared to the original SA/GL/PAm hydrogel (Fig. 6b). Two distinctive features are readily apparent. Firstly, the modified hydrogel showcases differences in the diameter of its macropores. This variation in pore size is a significant structural alteration resulting from the hydrolysis process, contributing to its enhanced water-absorbing capabilities. Secondly, the surface of the modified hydrogel is notably less smooth when compared to the original SA/GL/PAm hydrogel. This textured surface serves as an advantageous attribute for increased water absorption. The irregularities and microstructures on the hydrogel’s surface provide more sites for water molecules to interact with and be retained, ultimately enhancing the hydrogel’s ability to absorb and retain water efficiently. The SA/GL/PAm hydrogel and the UAH have different shapes, showing the hydrolysis process’s effect. This change in structure, along with the changes seen in the pores’ size and the surface’s texture, makes the modified hydrogel better at absorbing water. These insights are invaluable in understanding the underlying mechanisms that drive the hydrogel's enhanced performance and its potential applications in fields where efficient water management is essential. Also, Figure 6c shows the network structure of UAH after hydrolysis modification. The ability to retain a porous structure is crucial for the hydrogel’s effectiveness in applications such as soil moisture retention, indicating its potential for prolonged and efficient water management in agricultural settings.

### Water retention before and after SA/GL/PAm hydrolysis

**Figure 7:**
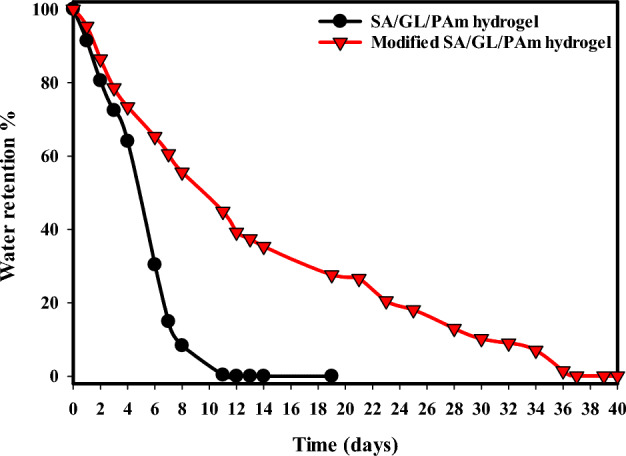
Water retention of SA/GL/PAm hydrogel and UAH at 30 °C.

Figure 7 shows an analysis of water retention that clearly shows how the SA/GL/PAm hydrogel and the UAH are very different in how well they can absorb water. This critical information is pivotal in understanding the hydrogel’s potential in various applications where efficient water management is essential. For the SA/GL/PAm hydrogel, the water retention percentage (WR%) reveals a significant decrease over time. After just 5 days, there is an approximately 50% reduction in WR%, which further decreases to 75% of the initial water weight after 6 days. Notably, within 11 days, the SA/GL/PAm hydrogel loses all its water content, highlighting its limited water-retaining capacity. This is a crucial factor in applications requiring prolonged water retention. In stark contrast, the UAH demonstrates extraordinary water-retaining capabilities. Even after 9 days, there is only a 50% reduction in WR%, a rate much slower than that of the unmodified hydrogel. Remarkably, the modified hydrogel maintains its water content for an extended period, retaining water for up to 36 days. This substantial difference in water retention can be attributed to the high hydrophilic nature of the modified hydrogel, which is characterized by numerous hydrophilic groups that can readily form hydrogen bonds with water molecules. Its effective porosity and substantial surface area further contribute to its ability to absorb and retain a significant volume of water. These findings underscore the considerable impact of the hydrolysis process on the hydrogel’s water-absorbing properties. The UAH’s ability to retain water for an extended duration makes it an ideal candidate for applications where sustained water retention is crucial, such as agriculture in arid regions, horticulture, and environmental management. The ability to efficiently manage water resources through such advanced materials represents a critical step towards addressing water scarcity and enhancing sustainability in various sectors.

### Enhancing the morphological and yield of wheat plants using UAH under drought conditions

**Figure 8:**
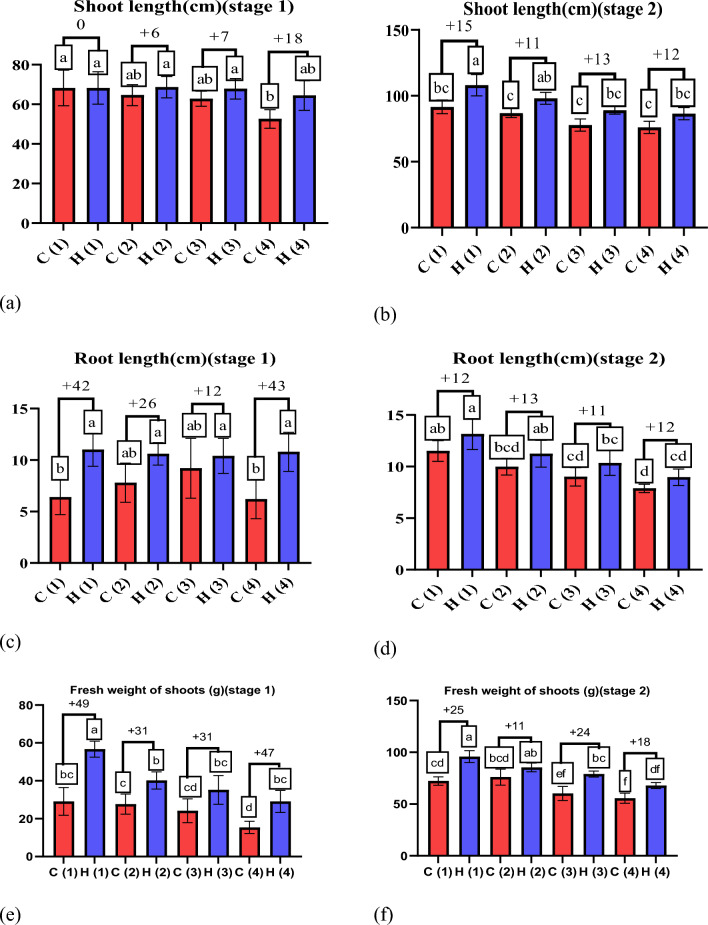

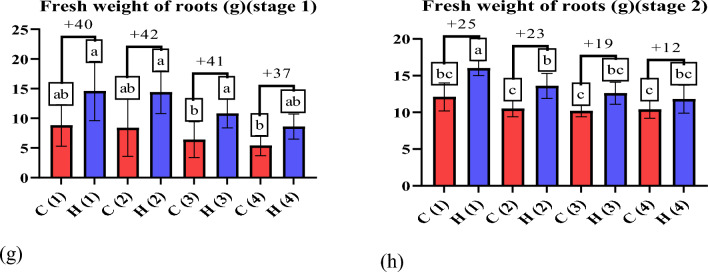
The effect of UAH and controlled (H and C) samples on (**a**,** b**) shoot length (**c**,** d**) root length (**e**, **f**) fresh weight of shoots, and (**g**, **h**) fresh weight of roots at stages (1, 2), respectively.

**Figure 9:**
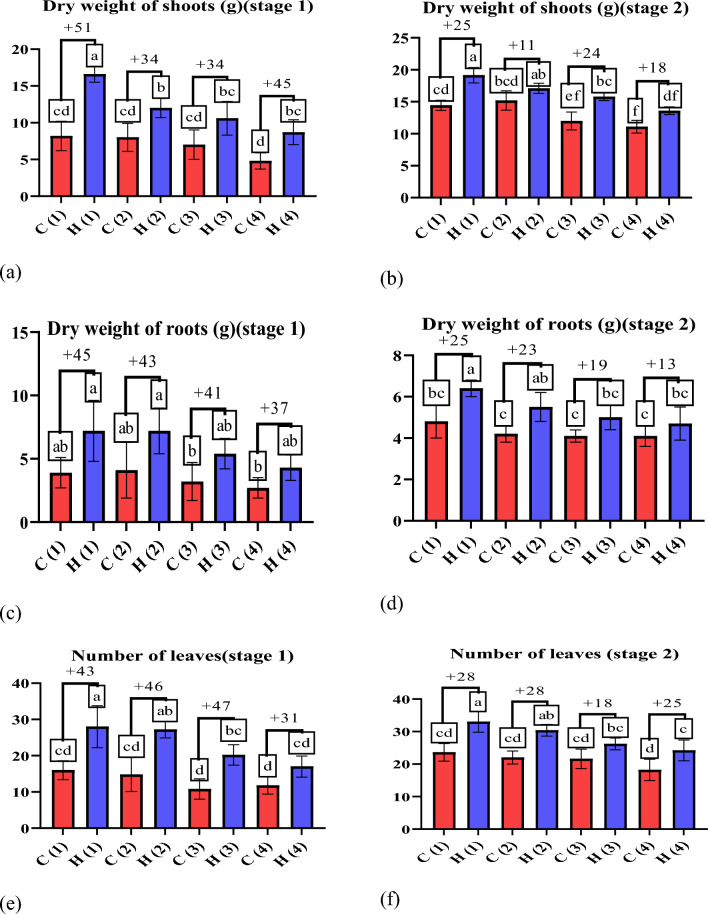
The effect of UAH and controlled (H and C) samples on (**a**,** b**) the dry weight of shoots, (**c**,** d**) the dry weight of roots, and (**e**,**f**) the number of leaves at stages (1, 2), respectively.

**Figure 10:**
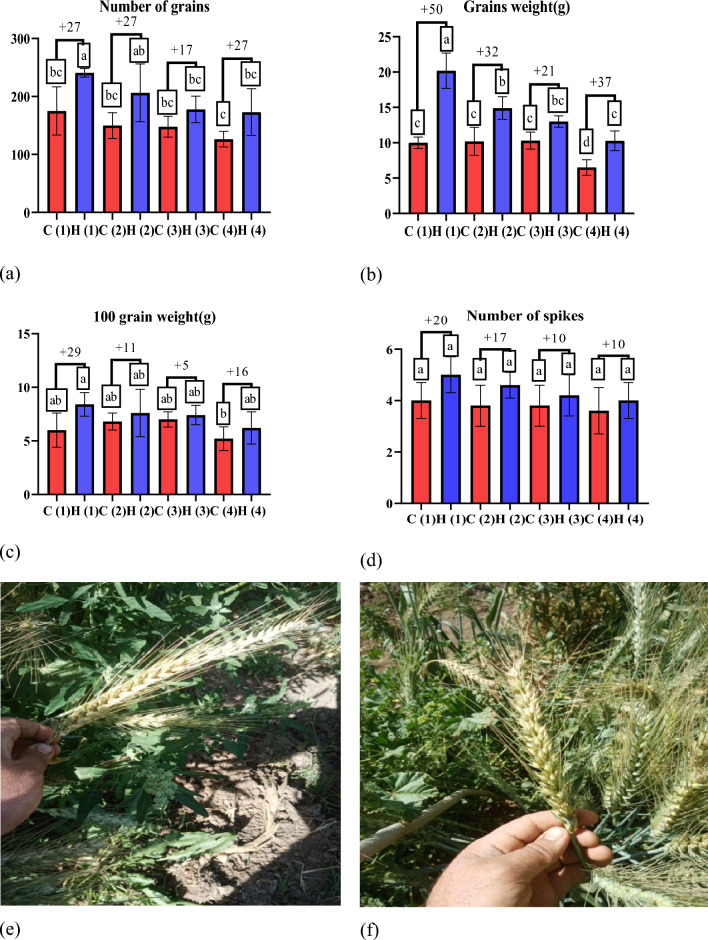
The effect of UAH and controlled (H and C) samples on the number of grains, grain weight, 100-grain weight, and number of spikes (**a**–**d**), respectively. Photographs of wheat spikes C and H (**e**,** f**), respectively.

Drought stress is a condition in which plants lack water or dehydration, negatively affecting their growth, development and overall health^37^. Hydrogels can increase biomass production in wheat plants, resulting in taller plants with more leaves^38^. The data presented in Fig. 8 illustrates the positive impact of ultra-absorbent hydrogel (UAH) on shoot length (g) under varying stress conditions. In the first stage (after 45 days), UAH demonstrated a notable enhancement in shoot length by 0%, 6%, 7%, and 18% at stress levels of 0%, 25%, 50%, and 100%, respectively (Fig. 8a). Similarly, in the second stage (after 85 days) (Fig. 8b), UAH exhibited a positive effect on shoot length with enhancements of 15%, 11%, 13%, and 12% at stress levels of 0%, 25%, 50%, and 100%, respectively, compared to control groups. This data underscores the capacity of hydrogels, such as UAH, to positively influence root growth and development by ensuring a consistent water supply, particularly under varying stress conditions^39^. The observed positive impact of UAH on shoot length also extends to root development, fostering healthier root systems with increased strength and extensiveness. As depicted in the data presented in Fig. 8c, UAH exhibits a substantial enhancement in root length by 42%, 26%, 12%, and 43% under stress levels of 0%, 25%, 50%, and 100%, respectively. Furthermore, at stage two (Fig. 8d), UAH demonstrates a significant difference in root length with enhancements of 12%, 13%, 11%, and 12%, respectively. This consistent improvement in root length underscores the beneficial influence of UAH on the overall health and development of wheat plants, contributing to taller and more robust growth^40,41^, As depicted in Fig. 8e–h the application of UAH to wheat plants resulted in a notable enhancement in shoot fresh weight. The data illustrates that the use of UAH positively influences the overall weight of the shoots, indicating a potential for increased biomass and healthier plant development. The precise values and details provided in the figure can offer a more comprehensive understanding of the extent of this enhancement in shoot fresh weight attributed to the application of UAH^42^. The positive impact of the hydrogel on wheat plants is evident in the data presented in Fig. 9a, b showcasing the enhancement in wheat dry weight shoots and roots. Particularly noteworthy is the positive influence observed in shoot dry weight. In stage one, the hydrogel application increased by 51%, 34%, 34%, and 45% at stress levels of 0%, 25%, 50%, and 100%, respectively. Similarly, in stage two, the hydrogel demonstrated a positive effect on shoot dry weight with enhancements of 25%, 11%, 24%, and 18% at stress levels of 0%, 25%, 50%, and 100%, respectively. These findings emphasize the hydrogel’s potential to significantly improve fresh and dry weight in wheat plants, particularly in adverse stress conditions. Hydrogels can protect wheat crops from the detrimental effects of drought^43^. Acting as a water reservoir, hydrogels give plants access to moisture during dry periods, effectively minimizing yield losses caused by drought-induced stress^44^. Also, dry weight of roots as shown in Fig. 9c, d in stage one, the hydrogel application increased by 45%, 43%, 41%, and 37% at stress levels of 0%, 25%, 50%, and 100%, respectively. Similarly, in stage two, the hydrogel demonstrated a positive effect on roots dry weight with enhancements of 25%, 23%, 19%, and 13% at stress levels of 0%, 25%, 50%, and 100%, respectively. Figure 9e, f show the positive impact of UAH on number of leaves under varying stress conditions. In the first stage, UAH demonstrated a notable enhancement in number of leaves by 43%, 46%, 47%, and 31% at different stress levels. In second stage, the hydrogel demonstrated a positive effect on number of leaves with enhancements of 28%, 28%, 18%, and 25% at stress levels of 0%, 25%, 50%, and 100%, respectively. As displayed in Fig. 10, it is clear that UAH had superiority of all yield parameters; the number of grains in Fig. 10a increased by using UAH; there was also an improvement in grain weight in Fig. 10b by 50, 32, 21, and 37% at 0, 25, 50, and 100% stress, respectively; the 100-grain weight in Fig. 10c increased at all stress levels of UAH treatments; and the number of spikes increased in the presence of UAH (Fig. 10d). Photographs of wheat spikes, as depicted in Fig. 10e, f provide visual evidence confirming the disparity between wheat plants cultivated with and without UAH presence. Also, Fig. 2s show the size and shape of the spike of wheat for four groups samples.

### Enhancing nitrogen management and metabolites in wheat plants using UAH

**Figure 11:**
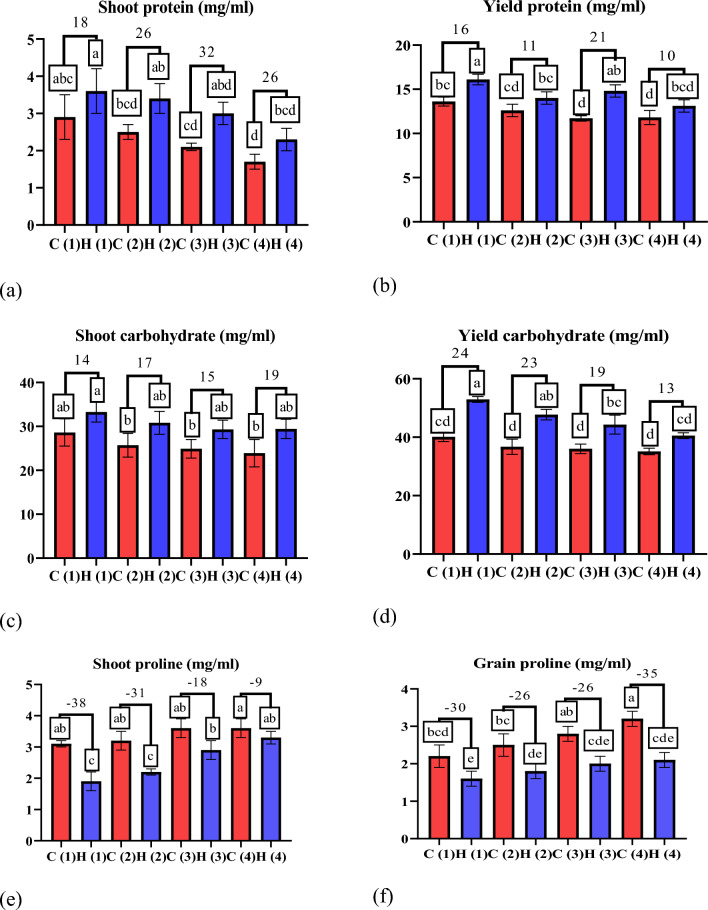

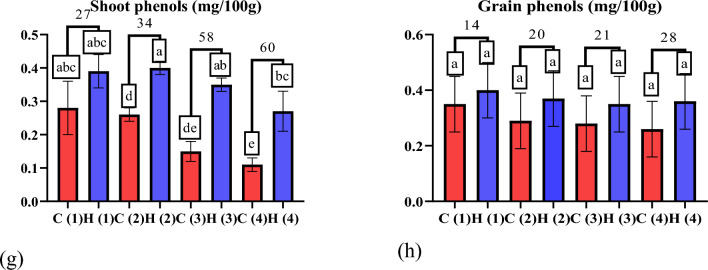
The effect of UAH and controlled (H and C) samples on (**a**,** b**) protein (mg/ml), (**c**,** d**) carbohydrate (mg/ml), (**e**,** f**) proline (mg/ml), and (**g**,** h**) phenol (mg/100 g).

As evident in Fig. 11a, b UAH demonstrated promising effects on wheat plants subjected to varying drought stress levels. The considerable increase in shoot protein content by 18%, 26%, 32%, and 26% at stress levels of 0%, 25%, 50%, and 100%, respectively, along with the corresponding increment in grain protein content by 16%, 11%, 21%, and 10%, directly supports the notion that UAH positively influences the nitrogen content of the plant. This impact is crucial for synthesizing proteins, underscoring the potential benefits of UAH in promoting healthier plant growth under stress conditions. These findings corroborate earlier research^45^, which also recognized the benefits of hydrogels in enhancing plant protein content. Hydrogels contribute to overall plant health and stimulate photosynthesis^46^, a fundamental process in which plants transform carbon dioxide and water into carbohydrates. As illustrated in Fig. 11c, d there is a notable increase in shoot carbohydrate content by 14%, 17%, 15%, and 19% at stress levels of 0%, 25%, 50%, and 100%, respectively. Similarly, the grain carbohydrate content experiences increment of 24%, 23%, 19%, and 13%. These findings suggest that ultra-absorbent hydrogel (UAH) has the potential to enhance photosynthesis, aligning with previous research. The observed surge in carbohydrate content further supports the idea that UAH positively influences the plant’s metabolic processes, contributing to improved overall plant health and resilience under varying stress conditions^46^. Moreover, plants often accumulate proline under drought stress conditions as a defensive mechanism to maintain cellular stress and osmotic balance^47^. The consistent water supply provided by hydrogels can alleviate drought stress and reduce the need for proline accumulation, which acts as an osmoprotectant in cells under biotic stress. The reduction in shoot proline content by 38%, 31%, 18%, and 9% and the decrease in grain proline content by 30%, 26%, 26%, and 35%, as evident in Fig. 11e, f underscore the effectiveness of UAH in mitigating drought stress. This is consistent with previous research^48^, which also demonstrated a decrease in proline content in sunflower plants by applying hydrogels. Phenolic compounds, known for their various functions in plants, such as defense mechanisms, stress responses, and antioxidant activities, were also impacted by UAH. Contrary to some previous findings^49^, the data in Fig. 11g, h revealed an increase in phenolic compounds at all stress levels compared to control treatments. This variation could be elucidated by allocating more resources towards secondary metabolites, specifically phenolic compounds when plants undergo mild drought stress. Such resource allocation may enhance the plant's stress response and defense mechanisms. This discrepancy emphasizes the complexity of plant responses to stress conditions, where factors such as stress severity and the specific metabolic pathways activated can lead to varied outcomes in secondary metabolite production.

### Enhancing pigment eliminated the oxidative stress in wheat plants using UAH

Chlorophyll is an essential chemical found in the chloroplasts of plant cells and other photosynthetic organisms, such as algae and some bacteria^50^. Hydrogels play a crucial role in maintaining consistent soil moisture levels, a factor essential for photosynthesis, and preserving adequate chlorophyll levels. This aligns with the outcomes presented in Fig. 3s, where UAH positively impacted chlorophyll content. In Fig. 3s (a, b) UAH enhanced chlorophyll a during stage 1 by 29%, 20%, 10%, and 14%, and during stage 2 by 24%, 15%, 9%, and 7% at stress levels of 0%, 25%, 50%, and 100%, respectively. Similarly, in Fig. 3s (c, d), UAH increased chlorophyll b during stage 1 by 38%, 28%, 15%, and 37% and during stage 2 by 15%, 21%, 12%, and 31% at stress levels of 0%, 25%, 50%, and 100%, respectively. Moreover, in Fig. 3s (e, f), UAH demonstrated superiority in total Chlorophyll during stage 1 by 31%, 22%, 11%, and 20% and during stage 2 by 21%, 17%, 10%, and 14%, at stress levels of 0%, 25%, 50%, and 100%, respectively. Lastly, in Fig. 3s (g, h), UAH enhanced carotenoid content during stage 1 by 27%, 14%, 26%, and 25% and during stage 2 by 24%, 14%, 24%, and 27% at stress levels of 0%, 25%, 50%, and 100%, respectively. These findings underscore the positive influence of UAH on the photosynthetic pigments crucial for plant growth and stress resilience. These results align with^51^, which revealed that superabsorbent hydrogel enhanced the chlorophyll and carotenoid content of sunflowers.

Catalase is an enzyme involved in the detoxification of reactive oxygen species (ROS) produced under stress conditions^52^. Hydrogel can reduce catalase enzyme content in plants by maintaining soil moisture and reducing drought stress. In line with this, the results displayed in Fig. 4s (a, b) showed that UAH reduced catalase enzyme stage (1) by 29, 22, 29, and 38%, and stage (2) by 26, 32, 19, and 16%, at 0, 25, 50, and 100%, respectively. These results were verified by previous research on sunflowers^13^. The Peroxidase enzyme is involved in a variety of physiological processes in plants, including creating defenses against oxidative stress^53^. Hydrogel can reduce peroxidase enzyme content by improving water retention in the root zone and reducing oxidative stress in plants. This goes with the results displayed in Fig. 4s (c, d), where UAH decreased peroxidase enzyme stage (1) by 19, 22, 25, and 24% and stage (2) by 31, 18, 16, and 30% at 0, 25, 50, and 100%, respectively. Polyphenol oxidase (PPO) is an enzyme found in plants that helps turn fruits and vegetables brown when they are cut or damaged^54^. Hydrogel can indirectly reduce PPO activity by limiting the occurrence of tissue damage and minimize physical damage to plant tissues by preventing excessive drying and wilting. The results displayed in Fig. 4s (e, f) revealed that UAH reduced polyphenol oxidase enzyme stage (1) by 9, 6, 10, and 12% and stage (2) by 15, 13, 23, and 25% at 0, 25, 50, and 100%, respectively. In line with these results^48^, hydrogels decrease the polyphenol oxidase enzyme in sunflower plants, One well-known function of hydrogen peroxide in plants is its role in responses to biological and abiotic stresses^55^. Hydrogels can reduce H_2_O_2_ by mitigating drought stress; the data displayed in Fig. 5 s (a, b) showed that UAH reduced H_2_O_2_ stage (1) by 16, 10, 11, and 17%, and stage (2) by 25, 16, 21, and 22% at 0, 25, 50, and 100%, respectively. This results goes with the previous research on maize plant^7^ which indicated that UAH reduce hydrogen peroxide in maize plant, malondialdehyde (MDA) is a marker of lipid peroxidation and oxidative stress in plant cells^56^, MDA can accumulate in plant tissues when the cells undergo oxidative damage^57^, hydrogel can reduce MDA by regulating temperature in the root zone of plants which plays a role in the rate of oxidative reactions, the results displayed in Fig. 5s (c and d) indicated that UAH reduced MDA stage (1) by 45, 23, 34 and 38% and stage (2) by 21, 22, 18 and 36% at 0, 25, 50 and 100% respectively, in line with the results^58^ revealed that hydrogel reduced MDA content under stress condition.

## Conclusion

The study developed an ultra-absorbent biopolymer hydrogel using sodium alginate, gelatin, and polyacrylamide for efficient water management and nitrogen regulation in wheat under drought stress. The hydrogel was synthesized by gamma irradiation and modified through hydrolysis to achieve remarkably high water absorption (1293% swelling) and extended water retention (36 days). Characterization confirmed chemical transformations enabling enhanced moisture retention. Application to wheat crops revealed significantly improved growth metrics, grain yield, protein, and carbohydrates under extreme 100% drought conditions compared to controls. Chlorophyll levels increased up to 31% with hydrogel treatment, while antioxidant enzyme activity declined, reducing oxidative damage. The biopolymer hydrogel demonstrated outstanding potential for water conservation and controlled nutrient release to enhance wheat yield and nutrition under moisture limitations. The technology could be extended to various water-intensive crops and optimized for diverse agro-climates. Further analysis of nutrient release kinetics and soil health impacts is warranted.

### Supplementary Information

Supplementary Information.

## Data Availability

All data generated or analyzed during this study available from the corresponding author on request.

## References

[1] Asseng, S. *et al.* Can Egypt become self-sufficient in wheat?. *Environ. Res. Lett.* **13**, 094012 (2018). 10.1088/1748-9326/aada50

[2] Sharaf, A. Effect of phosphorus, zinc and sulphur application on the growth characters, P and Zn uptake, yield components of wheat plant grown on calcareous soil. *J. Soil Sci. Agric. Eng.* **33**, 6265–6278 (2008).

[3] Yigezu, Y. A. *et al.* Food losses and wastage along the wheat value chain in Egypt and their implications on food and energy security, natural resources, and the environment. *Sustainability* **13**, 10011 (2021). 10.3390/su131810011

[4] Viviroli, D. *et al.* Climate change and mountain water resources: Overview and recommendations for research, management and policy. *Hydrol. Earth Syst. Sci.* **15**, 471–504 (2011). 10.5194/hess-15-471-2011

[5] Patra, S. K. *et al.* Prospects of hydrogels in agriculture for enhancing crop and water productivity under water deficit condition. *Int. J. Polym. Sci.* **2022**, 1–15 (2022). 10.1155/2022/4914836

[6] Opoku, D. G. *Assessing the Use of Hydrogels to Harvest Atmospheric Water for Agriculture in Arid and Semi-Arid areas: A Thesis Presented in Partial Fulfilment of the Requirements for the Degree of Master of Environmental Management at Massey University* (Massey University, 2021).

[7] Ghobashy, M. M. *et al.* Radiation cross-linked ultra-absorbent hydrogel to rationalize irrigation water and fertilizer for maize planting in drought conditions. *Int. J. Biol. Macromol.* **252**, 126467 (2023). 37640186 10.1016/j.ijbiomac.2023.126467

[8] Ghobashy, M. M. *Hydrogels Based on Natural Polymers* 329–356 (Elsevier, 2020).

[9] Ghobashy, M. M. *et al.* Controlling radiation degradation of a CMC solution to optimize the swelling of acrylic acid hydrogel as water and fertilizer carriers. *Poly. Adv. Technol.* **32**, 514–524 (2021). 10.1002/pat.5105

[10] Ghobashy, M. M., Mousaa, I. M. & El-Sayyad, G. S. Radiation synthesis of urea/hydrogel core shells coated with three different natural oils via a layer-by-layer approach: An investigation of their slow release and effects on plant growth-promoting rhizobacteria. *Prog. Org. Coat.* **151**, 106022–106034 (2021). 10.1016/j.porgcoat.2020.106022

[11] Hussain, S. B., Karagiannis, E., Manzoor, M. & Ziogas, V. From stress to success: Harnessing technological advancements to overcome climate change impacts in citriculture. *Crit. Rev. Plant Sci.* **42**, 345–363 (2023). 10.1080/07352689.2023.2248438

[12] Deng, W. *et al.* Cellulose nanofibril as a crosslinker to reinforce the sodium alginate/chitosan hydrogels. *Int. J. Biol. Macromol.* **189**, 890–899 (2021). 34455006 10.1016/j.ijbiomac.2021.08.172

[13] Alkhursani, S. A. *et al.* Application of nano-inspired scaffolds-based biopolymer hydrogel for bone and periodontal tissue regeneration. *Polymers* **14**, 3791 (2022). 36145936 10.3390/polym14183791 PMC9504130

[14] Tang, W.-J. *et al.* Preparation and properties of sodium alginate/gelatin hydrogel-microspheres. *J. Macromol. Sci. Part B* **63**, 146–160 (2023). 10.1080/00222348.2023.2254578

[15] Abd El-Sattar, N. E. A., El-Hddad, S. E. S. A., Ghobashy, M. M., Zaher, A. A. & El-Adl, K. Nanogel-mediated drug delivery system for anticancer agent: pH stimuli responsive poly (ethylene glycol/acrylic acid) nanogel prepared by gamma irradiation. *Bioorg. Chem.* **127**, 105972–105984 (2022). 35728290 10.1016/j.bioorg.2022.105972

[16] Ghobashy, M. M. *et al.* Radiation crosslinking of modifying super absorbent (polyacrylamide/gelatin) hydrogel as fertilizers carrier and soil conditioner. *J. Polym. Environ.* **26**, 3981–3994 (2018). 10.1007/s10924-018-1273-9

[17] Giraldo, J. D., Garrido-Miranda, K. A. & Schoebitz, M. Chitin and its derivatives: Functional biopolymers for developing bioproducts for sustainable agriculture—A reality?. *Carbohyd. Polym.* **299**, 120196 (2023). 10.1016/j.carbpol.2022.120196 36876809

[18] Ghani, S. A. A. *et al*. Characterization and distribution of plastic particles along Alexandria beaches, Mediterranean Coast of Egypt, using microscopy and thermal analysis techniques. *Sci. Total Environ.* **834**, 155363–155373 (2022). 35460789 10.1016/j.scitotenv.2022.155363

[19] El-Sayed, A. A. M. *et al*. Microplastics contamination in commercial fish from Alexandria City, the Mediterranean Coast of Egypt. *Environ. Pollut.* **313**, 120044–120052 (2022). 36064058 10.1016/j.envpol.2022.120044

[20] Watson, C. A. *et al.* Grain legume production and use in European agricultural systems. *Adv. Agron.* **144**, 235–303 (2017). 10.1016/bs.agron.2017.03.003

[21] Gayeda, H. M. *et al.* Surface modification of composite polyamide reverse osmosis membrane by irradiated chitosan and TiO. *Desalin. Water Treat* **1**, 9 (2019).

[22] Maziad, N. A. *et al.* Radiation synthesis and characterization of super absorbent hydrogels for controlled release of some agrochemicals. *J. Radioanal. Nucl. Chem.* **307**, 513–521 (2016). 10.1007/s10967-015-4201-7

[23] Gayed, H., Masry, B., Sayed, M. & Awadallah-F, A. Development of Fe_3_O_4_/polyvinylalcohol-nanocomposite hydrogel by chemical and irradiation approaches for Sb (III) sorption from acidic medium. *Nanotechnol. Environ. Eng.* **8**, 691–705 (2023). 10.1007/s41204-023-00316-x

[24] Gayed, H. M. & Ghobashy, M. M. Gamma irradiation-enhanced performance of waste LLDPE thermally transformed into advanced sponge-like material for oil decontamination. *Sci. Rep.* **13**, 19222. 10.1038/s41598-023-46194-w (2023). 37932301 10.1038/s41598-023-46194-w PMC10628294

[25] Gayed, H. M. & Ghobashy, M. M. Fabrication of jelly like material from rLLDPE by a binary approach based on gamma irradiation and thermal processing for oil remediation. *Discov. Appl. Sci.* **6**, 1–17 (2024). 10.1007/s42452-024-05755-y

[26] Ghobashy, M. M. & Gayed, H. M. Thermal conversion of irradiated LLDPE waste into sustainable sponge-like compounds: A novel approach for efficient trace-level oil–water removal. *Sci. Rep.* **14**, 4833 (2024). 38413688 10.1038/s41598-024-55401-1 PMC10899568

[27] Khan, M. H. U. *et al.* Applications of artificial intelligence in climate-resilient smart-crop breeding. *J. Radioanal. Nucl. Chem.* **23**, 11156 (2022). 10.3390/ijms231911156 PMC9570104 36232455

[28] de Aguiar, K. L. N. P., de Oliveira, P. F. & Mansur, C. R. E. A comprehensive review of in situ polymer hydrogels for conformance control of oil reservoirs. *Oil Gas Sci. Technol. Rev. d’IFP Energ. Nouv.* **75**, 8 (2020). 10.2516/ogst/2019067

[29] Amir, Z. *et al.* The retardation of polyacrylamide by ammonium chloride in high-salinity and high-temperature conditions: Molecular analysis. *Polym. Bull.* **77**, 5469–5487 (2020). 10.1007/s00289-019-03023-3

[30] Taubner, T., Marounek, M. & Synytsya, A. Preparation and characterization of amidated derivatives of alginic acid. *Int. J. Biol. Macromol.* **103**, 202–207 (2017). 28526341 10.1016/j.ijbiomac.2017.05.070

[31] Gil, E. S. *Stimuli-Responsive Protein-Based Hydrogels by Utilizing β-Sheet Conformation of Silk Fibroin as Cross-Links* (North Carolina State University, 2005).

[32] Basu, T., Bhutani, U. & Majumdar, S. Cross-linker-free sodium alginate and gelatin hydrogels: A multiscale biomaterial design framework. *J. Mater. Chem. B* **10**, 3614–3623 (2022). 35507082 10.1039/D2TB00028H

[33] Ghobashy, M. M. & Bassioni, G. pH stimuli-responsive poly (acrylamide-co-sodium alginate) hydrogels prepared by γ-radiation for improved compressive strength of concrete. *Adv. Polym. Technol.* **37**, 2123–2133 (2018). 10.1002/adv.21870

[34] Demeter, M. *et al.* Network structure studies on γ–irradiated collagen–PVP superabsorbent hydrogels. *Radiat. Phys. Chem.* **131**, 51–59 (2017). 10.1016/j.radphyschem.2016.09.029

[35] El-Rehim, H. A. A., Hegazy, E. S. A. & El-Mohdy, H. L. A. Radiation synthesis of hydrogels to enhance sandy soils water retention and increase plant performance. *J. Appl. Polym. Sci.* **93**, 1360–1371 (2004). 10.1002/app.20571

[36] Gharekhani, H., Olad, A., Mirmohseni, A. & Bybordi, A. Superabsorbent hydrogel made of NaAlg-g-poly (AA-co-AAm) and rice husk ash: Synthesis, characterization, and swelling kinetic studies. *Carbohyd. Polym.* **168**, 1–13 (2017). 10.1016/j.carbpol.2017.03.047 28457428

[37] Wahab, A. *et al.* Plants’ physio-biochemical and phyto-hormonal responses to alleviate the adverse effects of drought stress: A comprehensive review. *Plants* **11**, 1620 (2022). 35807572 10.3390/plants11131620 PMC9269229

[38] Rajanna, G. *et al.* Biopolymeric superabsorbent hydrogels enhance crop and water productivity of soybean–wheat system in Indo-Gangetic plains of India. *Sci. Rep.* **12**, 11955 (2022). 35831395 10.1038/s41598-022-16049-x PMC9279312

[39] Teng, Z. *et al.* Agarose hydrogel composite supports microgreen cultivation with enhanced porosity and continuous water supply under terrestrial and microgravitational conditions. *Int. J. Biol. Macromol.* **220**, 135–146 (2022). 35963353 10.1016/j.ijbiomac.2022.08.046

[40] Paswan, M., Patel, S., Prajapati, V. & Dholakiya, B. Z. Preparation and characterization of slow-release fertilizers loaded guar gum-g-poly methylmethacrylate-cl-polylactic acid (Gg-g-PMMA-cl-PLA) hydrogel and its effect on wheat growth. *Int. J. Biol. Macromol.* **253**, 126979 (2023). 37739290 10.1016/j.ijbiomac.2023.126979

[41] Munns, R. Comparative physiology of salt and water stress. *Plant Cell Environ.* **25**, 239–250 (2002). 11841667 10.1046/j.0016-8025.2001.00808.x

[42] Shahid, S. A., Qidwai, A. A., Anwar, F., Ullah, I. & Rashid, U. Improvement in the water retention characteristics of sandy loam soil using a newly synthesized poly (acrylamide-co-acrylic acid)/AlZnFe_2_O_4_ superabsorbent hydrogel nanocomposite material. *Molecules* **17**, 9397–9412 (2012). 22864245 10.3390/molecules17089397 PMC6268066

[43] Ahluwalia, O., Singh, P. C. & Bhatia, R. A review on drought stress in plants: Implications, mitigation and the role of plant growth promoting rhizobacteria. *Resour. Environ. Sustain.* **5**, 100032 (2021).

[44] Elshafie, H. S. & Camele, I. Applications of absorbent polymers for sustainable plant protection and crop yield. *Sustainability* **13**, 3253 (2021). 10.3390/su13063253

[45] Singh, A., Singh, A. K. & Aswin, C. Effect of hydrogel and thiourea on yield, quality and nutrient uptake of Indian mustard under moisture stress condition. *Res. Crops* **18**, 42–48 (2017). 10.5958/2348-7542.2017.00008.0

[46] Yang, W., Guo, S., Li, P., Song, R. & Yu, J. Foliar antitranspirant and soil superabsorbent hydrogel affect photosynthetic gas exchange and water use efficiency of maize grown under low rainfall conditions. *J. Sci. Food Agric.* **99**, 350–359 (2019). 29882362 10.1002/jsfa.9195

[47] Demiralay, M. Exogenous acetone O-(4-chlorophenylsulfonyl) oxime alleviates Cd stress-induced photosynthetic damage and oxidative stress by regulating the antioxidant defense mechanism in *Zea mays*. *Physiol. Mol. Biol. Plants* **28**, 2069–2083 (2022). 36573151 10.1007/s12298-022-01258-5 PMC9789276

[48] Salama, A. *et al.* Using cellulose-based hydrogel to alleviate the effect of drought stress of sunflower plant. *Egypt. J. Chem.* **66**, 355–362 (2023).

[49] Klein, M. & Poverenov, E. Natural biopolymer-based hydrogels for use in food and agriculture. *J. Science Food Agric.* **100**, 2337–2347 (2020). 10.1002/jsfa.10274 31960453

[50] Shabaka, S. *et al*. Prevalence and risk assessment of microplastics in the Nile Delta estuaries: “The Plastic Nile” revisited. *Sci. Total Environ.* **852**, 158446–158457 (2022). 36058336 10.1016/j.scitotenv.2022.158446

[51] Keshavarz, L., Farahbakhsh, H. & Golkar, P. Effect of hydrogel and irrigation regimes on chlorophyll content, nitrogen and some growth indices and yield of forage millet (*Pennisetum glaucum* L.). *Isfahan Univ. Technol. J. Crop Prod. Process.* **3**, 147–161 (2013).

[52] Saed-Moucheshi, A., Shekoofa, A. & Pessarakli, M. Reactive oxygen species (ROS) generation and detoxifying in plants. *J. Plant Nutr.* **37**, 1573–1585 (2014). 10.1080/01904167.2013.868483

[53] Ahmad, S. *Oxidative Stress and Antioxidant Defenses in Biology* (Springer Science & Business Media, 2012).

[54] Singh, B. *et al.* Enzymatic browning of fruit and vegetables: A review. In *Enzymes in Food Technology: Improvements and Innovations* (ed. Kuddus, M.) 63–78 (Springer, 2018).

[55] Qiao, W., Li, C. & Fan, L.-M. Cross-talk between nitric oxide and hydrogen peroxide in plant responses to abiotic stresses. *Environ. Exp. Bot.* **100**, 84–93 (2014). 10.1016/j.envexpbot.2013.12.014

[56] Całyniuk, B. *et al.* in *Annales Academiae Medicae Silesiensis.* 224–228 (Śląski Uniwersytet Medyczny w Katowicach).

[57] Li, S. *et al.* Cadmium-induced oxidative stress, response of antioxidants and detection of intracellular cadmium in organs of moso bamboo (*Phyllostachys pubescens*) seedlings. *Chemosphere* **153**, 107–114 (2016). 27015570 10.1016/j.chemosphere.2016.02.062

[58] Du, F. *et al.* A novel biochar-based composite hydrogel for removing heavy metals in water and alleviating cadmium stress in tobacco seedlings. *Sci. Rep.* **13**, 15656 (2023). 37730828 10.1038/s41598-023-41946-0 PMC10511474

