# Supplementary material for: Radiation synthesis of sodium alginate/gelatin based ultra-absorbent hydrogel for efficient water and nitrogen management in wheat under drought stress
Source: Sci Rep. 2024 Aug 22;14:19463. doi: 10.1038/s41598-024-69333-3 (PMC11341720; doi:10.1038/s41598-024-69333-3)
Supplement: Supplementary file 1 — Supplementary Information. [file 41598_2024_69333_MOESM1_ESM.pdf]

Supplementary file

Radiation Synthesis of Sodium Alginate/Gelatin based Ultra-Absorbent Hydrogel for Efficient Water and Nitrogen Management in Wheat under Drought Stress

Mahmoud A. El-diehy[1], Ibrahim I. Farghal[1], Mohamed A. Amin[1], Mohamed mohamady Ghobashy*[2], Abdelatti I. Nowwar[1], H. M. Gayed*[2]

[1]Botany and Microbiology Department, Faculty of Science, Al-Azhar University, Cairo, Egypt

[2]Radiation Research of Polymer Chemistry Department, National Center for Radiation Research and Technology (NCRRT), Egyptian Atomic Energy Authority (EAEA), Cairo, Egypt.

1- Experimental

A. Swelling kinetic studies

In order to determine water diffusion mechanism of hydrogel samples, the swelling data obtained from the first 60% of the fractional water uptake were fitted with the following equation:

$$W_t/W_\infty = kt^n \quad (1)$$

Where W_t and W_∞ correspond to the water uptake capacity at time t (min) and at equilibrium, respectively. k is a proportionality constant, and n determines the diffusion mechanism of water molecules. For swellable hydrogel systems, when $n < 0.5$, the diffusion mechanism is the Fickian diffusion type. For the non-Fickian (anomalous diffusion) type, n is between 0.5 and 1.0, and for the case II diffusion mechanism (relaxation-controlled transport), n is 1.0. Also, for supercase II diffusion, n is greater than 1.0. The plot of Ln (W_t/W_∞) versus Ln (t) for prepared hydrogel gave the n value [1].

B. Evaluation of the Effect of ultra absorbent hydrogel (UAH) on the growth of (*Triticuma estivum*) under drought stress.

Seeds of *Triticuma estivum* Var. Sakkha 95 were supplied from Sakkha research center, Agriculture Ministry, Kafr el-sheikh, Egypt. The study was done at El Gharbia, Egypt in sandy loam soil. The soil Physiochemical (analysis in Ain Shams University, faculty of agriculture, Cairo, Egypt, in the arid land research and services center) was displayed in **Table 1s**. The experiment involved the application of 5 grams of UAH per square meter of soil surface. The UAH was thoroughly mixed with the soil prior to sowing the wheat seeds to ensure even distribution and optimal absorption as shown in **Fig. 1s (e)**. The treatment process is details in **Table 2s** and **Fig. 1s**, where soil was either treated with UAH (designated as "Hydrogel") or left untreated (referred to as "Control"). These treatments were administered at four different stress levels: 0%, 25%, 50%, and 100%. The treatments were as follows: Control at 0% stress (C1), Hydrogel at 0% stress (H1), Control at 25% stress (C2), Hydrogel at 25% stress (H2), Control at 50% stress (C3),

Hydrogel at 50% stress (H3), Control at 100% stress (C4), and Hydrogel at 100% stress (H4) To accommodate varying stress levels, watering frequencies were adjusted accordingly: 0% stress (standard irrigation every 20 days), 25% stress (every 25 days), 50% stress (every 30 days), and 100% stress (every 40 days). Plant samples of *Triticuma estivum* (common wheat) were collected after 45 (first stage) and 85 (second stage) days from sowing to evaluate their morphological and biochemical characteristics, morphological trait including shoot and root lengths, fresh and dry shoot and root weights, and the number of leaves. Additionally, biochemical analyses were performed, encompassing parameters such as chlorophyll a and b, total chlorophyll, carotenoids, shoot phenol, shoot proline, shoot protein, shoot carbohydrate, hydrogen peroxide, malondialdehyde (MDA), and antioxidant enzyme contents. After 160 days, yield samples were collected for determination of number of spines, grains weight, 100 grain weight, number of grains, and contents of grain carbohydrates, proline, protein and phenolic compounds Samples were taken from five selected plants for each treatment to assess root length, new shoot weight, number of leaves and yield These measurements served to measure plant growth and development under different treatment conditions, and provided insight into treatment efficacy who introduced plant growth and yield. Growth measurements and biochemical analyzes under controlled conditions are discussed in details.

Table 1 Physiochemical estimation of the soil.

**Soil Texture**	**Sand (%) > 200 - 20 µm**	**Silt (%) 20 - 2 µm**	**Clay (%) < 2 µm**
Sandy loam	64.72	21.00	14.28

**CaCO3 %**	**Cations meq / l**				**Anions meq / l**			**ECe (dS/m)**	**PH at 1:2.5**
	**K+**	**Na+**	**Mg++**	**Ca++**	**Cl-**	**HCO3-**	**CO3--**		
4.20	0.39	8.89	3.45	5.00	8.25	4.95	*	1.90	7.67

**Conc. (mg/kg soil)**						
**N**	**K**	**P**	**Cu**	**Fe**	**Mn**	**Zn**
34.44	217.40	15.80	5.02	17.00	0.81	4.55

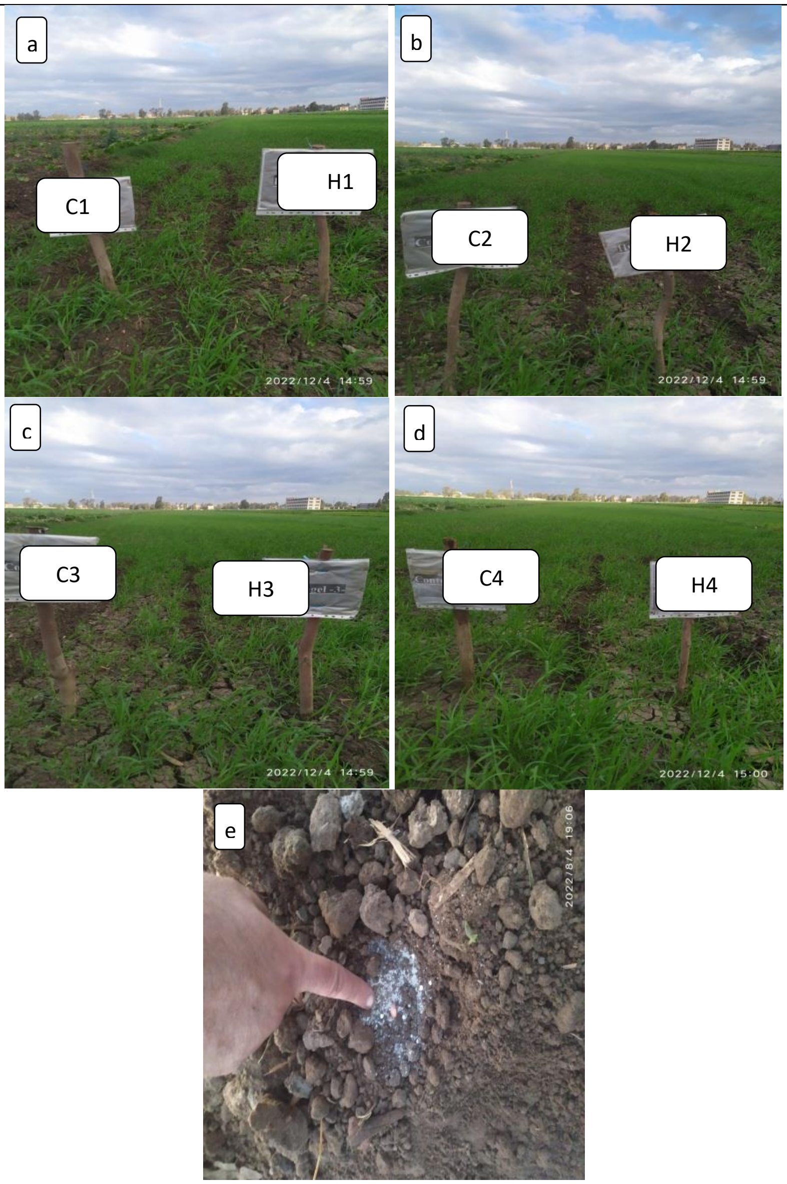

Fig. 1s Application of UAH on *Triticuma estivum* (common wheat) at different stress levels (0% (1), 25% (2), 50% (3) and 100% (4)) the control sample (without hydrogel) represented by (C) letter and while the hydrogel-enhanced sample represented by (H) letter.

Table 2s

Key words of treatments

Irrigation Time (days)	Control	Hydrogel	Water stress (%)
20	C1	H1	0
25	C2	H2	25

**30**	**C3**	**H3**	**50**
**40**	**C4**	**H4**	**100**

spike morphology of wheat through using hydrogel as a soil conditioner

We conducted an experiment to examine the spike morphology of wheat with hydrogel as a soil conditioner (**Figure 2s**, **Table 2s**). Under drought conditions, the average spikelet density (spikelet number per centimeter of spike length) was significantly decreased by four controlled samples. drought conditions led to a substantial decrease of spikelet density, while hydrogel led to a substantial increase in spikelet density and had a significant effect on total spikelet number per spike, spike length was significantly increased compared to control samples under drought conditions.

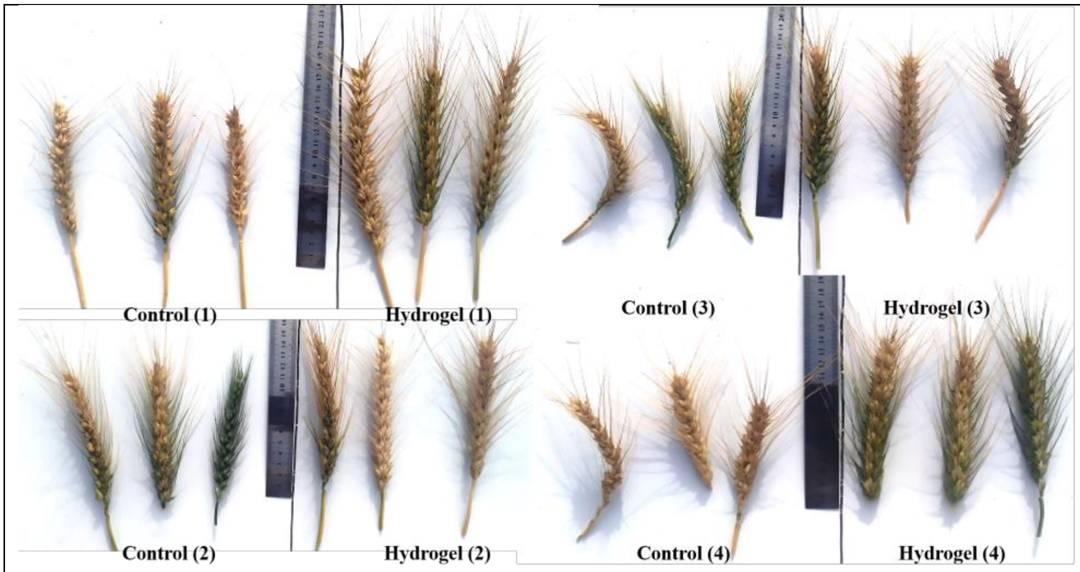

Figure 2s show the size and shape of the spike of wheat for four groups samples

C. Identification of pigments and carotenoids

In 1966, one gram of fresh green roots was weighed and then finely minced using the method described by [2]. Plant pigments were extracted by grinding root pieces in a mixture containing 100

ml of 80% acetone. The resulting mixture was then filtered, and the filtrate was carefully transferred to a 100 ml volumetric flask. The volume was adjusted to 100 ml with 80% acetone. Optical density measurements were obtained at two specific wavelengths, 649 nm and 665 nm. You can use the following equations to calculate the amounts of chlorophylls a and b, as well as their totals in plant tissues.

Chlorophyll a (Mg/g tissue) = 11.63 (A665) - 2.39 (A649).

Chlorophyll b (Mg/g root) = 20.11 (A649) - 5.18 (A665).

Total chlorophyll a + b (Mg/g tissue) = 6.45 (A665) + 17.72 (A649).

To estimate carotenoid chemical composition, the method described by [3] can be used:

Carotenoids (mg/g fresh weight) = (1000 * A470) – (1.82 * chlorophyll a) - (85.02 * chlorophyll b) / 198.

Please note that "(A)" represents the optical density.

D. Estimation of total phenolic compounds

The described method of [4] estimates the total phenolic compounds in the sample:

1- Take a small sample and fill it with distilled water in a volumetric flask.
2- Add Folin-Ciocalteau reagent and wait 1-8 minutes for reaction.
3- Add sodium carbonate solution to increase color.
4- Exchange the volume with water, wait 2 hours, and observe at 760 nm.
5- Construct a standard curve of gallic acid solution of known concentration.
6- Use the curve to calculate the phenolic content in mg GAE per gram of sample.
7- If the absorbance falls outside the curve, dilute and repeat the experiment until it fits within the range.

E. Calculation of free proline

To estimate free proline in plant tissues, [5]Methodology:

1- Grind 0.5 g of dried roots and mix with 10 ml of 3% sulfosalicylic acid.
2- Filter the mixture to obtain the extract.
3- Combine 2 ml of extract, 2 ml of acid ninhydrin and 2 ml of glacial acetic acid in a test tube.
4- Cook for 1 hour and stop the process in an ice bath.
5- Extract the contents of the tube with 4 ml of toluene and mix vigorously. Separate the top collar.
6- Measure the color absorbance at 520 nm using a UV-colorimeter with toluene as a blank.
7- Construct a standard curve using known concentrations of proline to determine concentrations based on the dry weight of the sample by the following equation:

$$\text{Mg/g proline} = \frac{(X)\ \text{ppm x ml Extract volume}}{2\ \text{x Sample dry weight x } 100}$$

E. Extraction and estimation of enzymes catalase, peroxidase and polyphenol oxidase.

❖ **Extraction**

Plant parts used to measure antioxidant enzymes (catalase, peroxidase, and polyphenol oxidase) were terminal shoots and young leaves. The procedure consisted of digesting 2 g of plant pellets with 10 ml of pH 6.8 phosphate buffer, followed by centrifugation at 20,000 rpm for 20 min at 20°C Clear supernatant containing the enzymes was collected as enzyme source [6].

❖ **Calculation of Catalase activities**

The reaction mixture comprises 10 ml in total, composed of 40 µl of enzyme extract and 9.96 ml of a phosphate buffer solution at pH 7.0 containing oxygenated water (prepared by adding 0.16 ml of 30% hydrogen peroxide to 100 ml of 50 mM phosphate buffer). Catalase activity was determined by measuring the change in H2O2 absorbance at 250 nm within 60 seconds using a UV-colorimeter. To establish a blank, the enzyme extract was substituted with buffer solution. In summary, the catalase activity was assessed by monitoring the decrease in H2O2 absorbance over 60 seconds in a reaction mixture containing the enzyme extract, phosphate buffer, and hydrogen peroxide, while a blank was prepared with buffer solution instead of the enzyme extract [7].

❖ **Peroxidase (POX) activities:**

Peroxidase activity was determined using a solution consisting of 10 ml, comprised of 5.8 ml of 50 mM phosphate buffer at pH 7.0, 200 µl of enzyme extract, and 2 ml of 20 mM H2O2, to which 2 ml of 20 mM pyrogallol was added. The rate of absorbance increase resulting from pyrogallol oxidation was measured using a UV-spectrophotometer at 470 nm over a 60-second period. A blank sample was prepared by substituting the enzyme extract with buffer. In the enzyme assay, the initial volume (at zero time) was considered as the blank. The enzyme activity was expressed as (Δ × T v × 60 min) / (t × v × F. Wt.), where Δ represents the change in absorbance during the incubation period, T v is the total volume of filtrate, t is the incubation time in minutes, v is the total volume of filtrate used for incubation, and F. Wt. is the fresh weight of the sample [8].

❖ **Polyphenol oxidase (PPO) activity:**

The activity of Polyphenol oxidase enzyme was calculated from the method described by [9]. The enzyme-substrate mixture consisted of 1.0 ml of enzyme extract, 1.0 ml of 0.2 M sodium phosphate

buffer at pH 7.0, 10 ml of 0.001 M catechol, and 3.0 ml of distilled water. The absorbance was measured at 495 nm, 60 seconds until and conversion was recorded in optical density. To determine enzyme activity, a blank was prepared by replacing the isolated enzyme with buffer solution. Enzyme activity, expressed as (Δ x T v x 60 min) / (t x v x F. Wt.), where Δ represents the difference in absorbance before and after incubation, T v being the total volume of the filtrate, t is the incubation time in minutes, v of the filtrate for incubation is the total volume, and F. Wt. the new weight of the sample. Results were statistically analyzed according to [10].

F. Extraction and Determination of Soluble Carbohydrates

❖ **Extraction**

The plant tissue after being dried at 60°C till a constant dry weight was ground to a fine powder. One gram of the powder to be analyzed was put in 100 ml capacity conical flask, to which 5 ml of 2% phenol water and 10 ml 30% trichloroacetic acid were added. The mixture was shaken and kept overnight before being filtered; the filtrate was made up to 50 ml [11].

❖ **Determination**

Contents of soluble carbohydrates were determined using anthrone technique according to [12], 10 ml of the extract was added to 2 g of activated charcoal and shaken vigorously for 15 minutes. The mixture was then filtered through Whatman No.1 filter paper in order to obtain a clear filtrate. Distilled water was added to this filtrate in bulk. Then, 2 ml of the dialyzed filtrate was placed in a test tube, and 4 ml of freshly prepared anthrone reagent (made up of 2 g of anthrone per 1 ml of pure 95% sulfuric acid) was added and then the test a was placed into a cold tube in a boiling water bath for 3 minutes. The resulting color was measured using a spectrophotometer with a wavelength of 620 nm. A blank mixture containing distilled water and anthrone reagent was used to calibrate the device to zero optical density (O.D.).

G. Extraction and Determination of Water-soluble Proteins

❖ **Extraction**

❖ The plant roots were dried at 60 °C until a constant dry weight was reached, then finely powdered to a fine powder and then one g of this dry powder was placed in a cone, where 2% phenol water was added; 5 ml mixed with 10 ml of distilled water. The reaction mixture was then filtered, and the resulting filtrate was adjusted with distilled water to a final volume of 50 ml.

❖ **Determination**

According to [13] using casein as a standard protein.

- **Reagents `**

Solution (A): 2% (Na_2CO_3) in 0.1 N (NaOH).

Solution (B): 0.5 g ($CuSO_4$) in 1% sodium potassium tartrate.

Solution (C): 50 ml of solution (A) were mixed with 1 ml of solution (B), mixing of the two solutions was done just before the protein determinations.

Solution (D): This solution was prepared by diluting folin reagent (BDH) with distilled water in the proportion of 1:3 (v/v).

- **Method**
- In a test tube, 1 ml of plant sample extracts intended for protein analysis was combined with 5 ml of solution (C). After thorough mixing, the tubes were left undisturbed for ten minutes. Then, 0.5 ml of solution (D) was rapidly added and mixed with the tube, and then left to stand for an additional 30 min and then the optical density (O.D.) of the resulting color was measured at a wavelength of 750 nm.
- **Enhancing pigment with eliminated the oxidative stress in wheat plant using UAH**

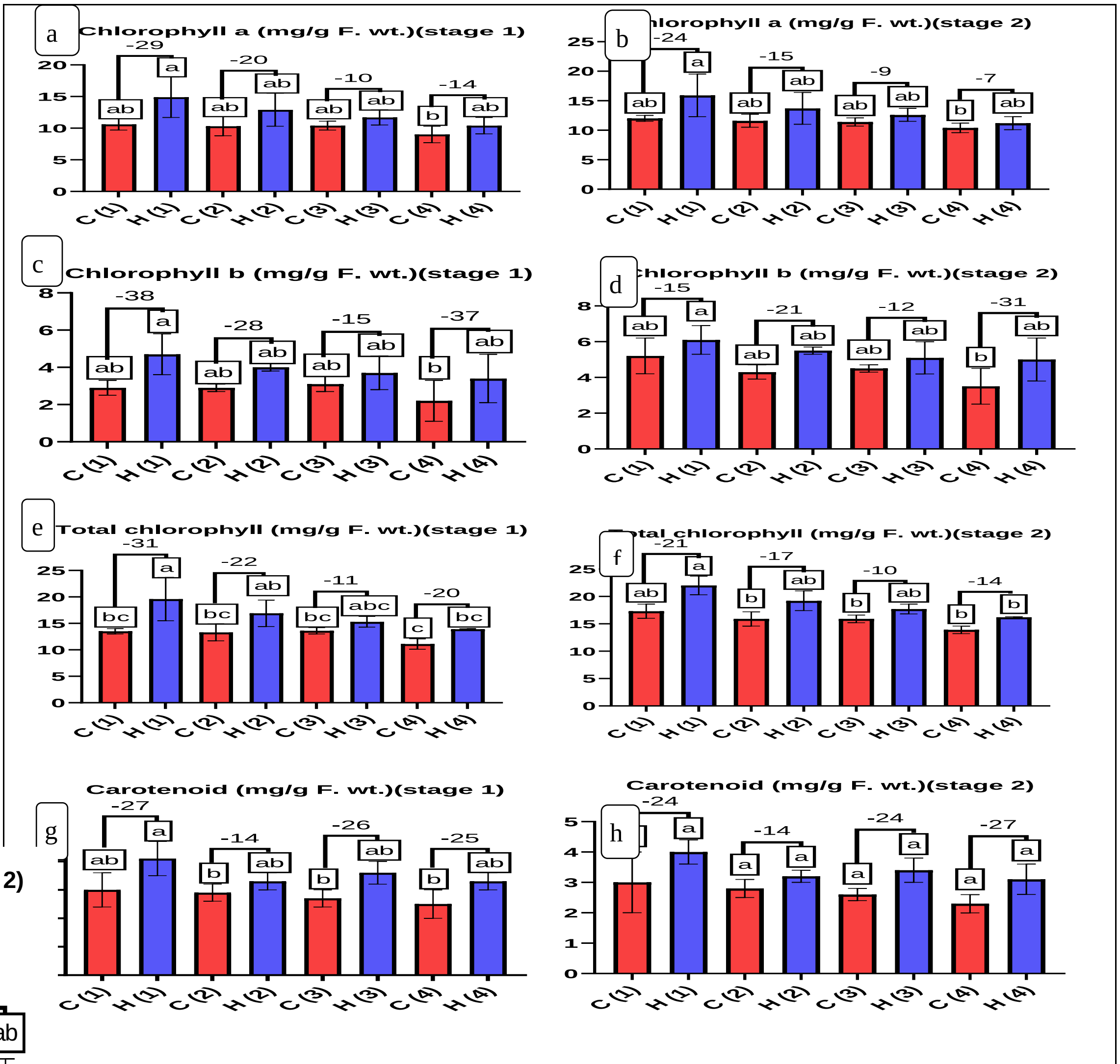

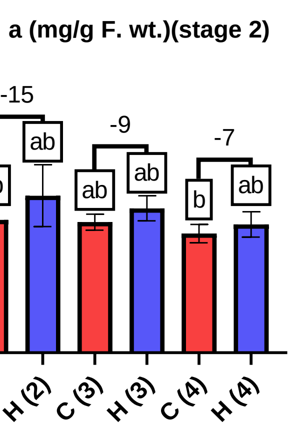

gure 3s: show the effect of UAH and controlled (H and C) samples on (a, b) orophyll a, (c, d) chlorophyll b, (e, f) total chlorophyll, and (g, h) carotenoid at stages 2), respectively.

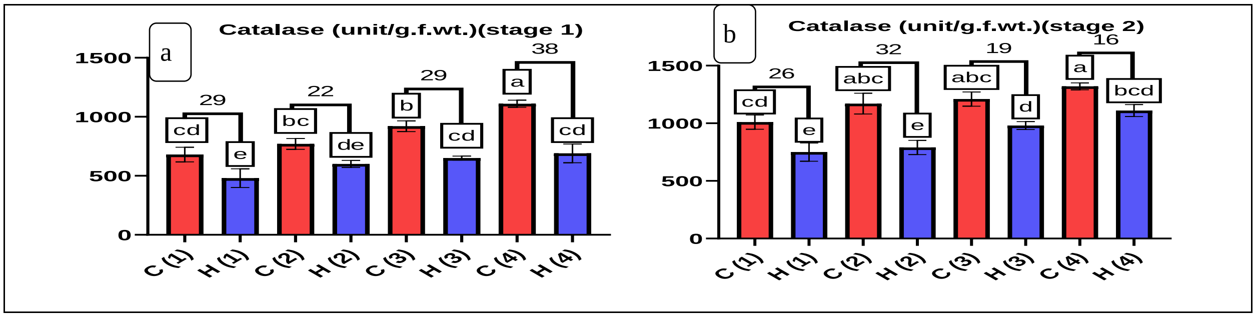

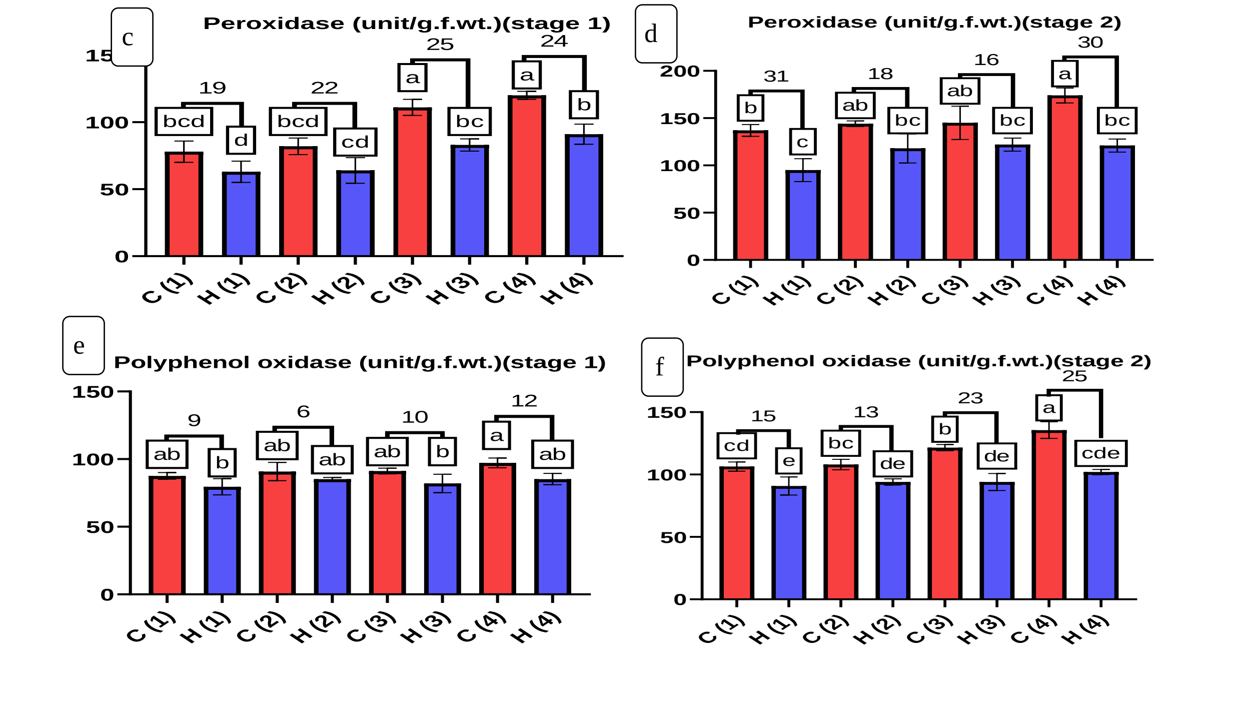

Figure 4s: show the effect of UAH and controlled (H and C) samples on (a, b) catalase, (c, d) peroxidase, and (e, f) polyphenol oxidase at stages (1, 2), respectively.

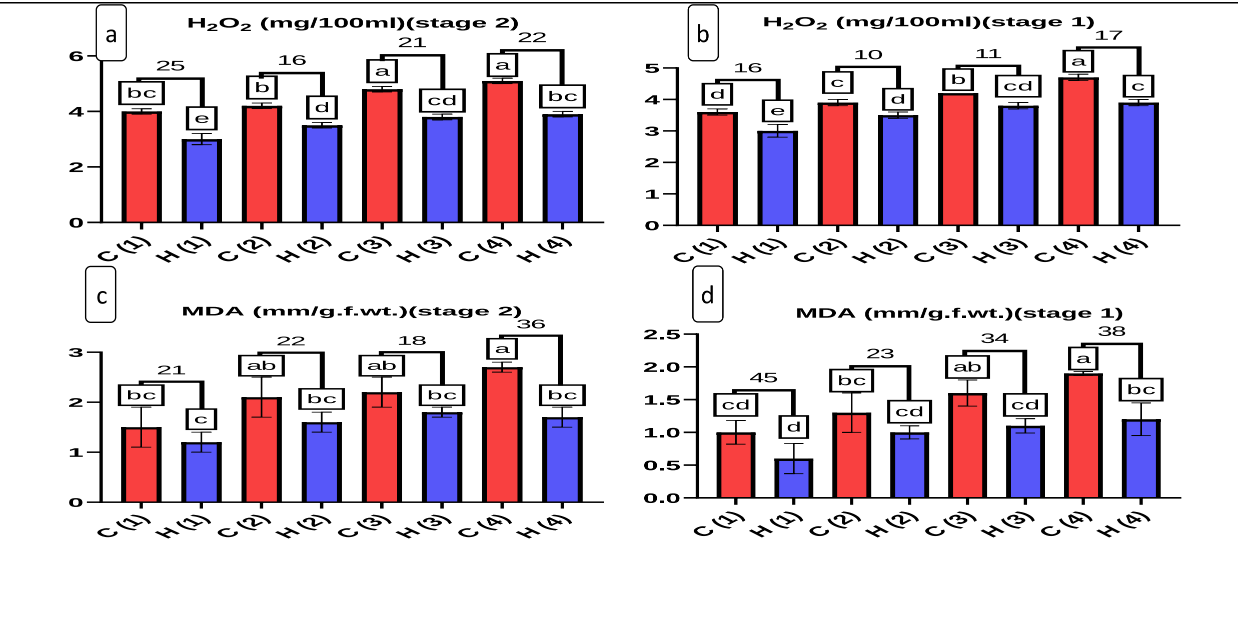

Figure 5s: show the effect of UAH and controlled (H and C) samples on (a, b) H_2O_2 and (c, d) MDA at stages (1, 2), respectively

❖ Refference

1 Gharekhani, H., Olad, A., Mirmohseni, A. & Bybordi, A. Superabsorbent hydrogel made of NaAlg-g-poly (AA-co-AAm) and rice husk ash: Synthesis, characterization, and swelling kinetic studies. *Carbohydrate polymers* **168**, 1-13 (2017).

2 Vernon, L. P. & Selly, G. R. (Acad. Press, New York, London, 1966).

3 Lichtenthaler, H. *et al.* Photosynthetic activity, chloroplast ultrastructure, and leaf characteristics of high-light and low-light plants and of sun and shade leaves. *Photosynthesis research* **2**, 115-141 (1981).

4 Singleton, V. L., Orthofer, R. & Lamuela-Raventós, R. M. in *Methods in enzymology* Vol. 299 152-178 (Elsevier, 1999).

5 Bates, L., Waldren, R. a. & Teare, I. Rapid determination of free proline for water-stress studies. *Plant and soil* **39**, 205-207 (1973).

6 Kong, F., Hu, W., Chao, S., Sang, W. & Wang, L. Physiological responses of the lichen Xanthoparmelia mexicana to oxidative stress of SO2. *Environmental and Experimental Botany* **42**, 201-209 (1999).

7 Aebi, H. in *Methods in enzymology* Vol. 105 121-126 (Elsevier, 1984).

8 Castillo, F. J., Penel, C. & Greppin, H. Peroxidase release induced by ozone in Sedum album leaves: involvement of Ca2+. *Plant physiology* **74**, 846-851 (1984).

9 Matta, A. & Dimond, A. Symptoms of Fusarium wilt in relation to quantity of fungus and enzyme activity in tomato stems. *Phytopathology* **53**, 574-& (1963).

10 Snedecor, G. W. & Cochran, W. G. Statistical methods, 8thEdn. *Ames: Iowa State Univ. Press Iowa* **54**, 71-82 (1989).

11 Said, A., Naguib, M. & Ramzy, M. Sucrose determination as a means of estimation of the draw back tax on exported Halawa Tehinia. *Bull. Fac. Sci. Cairo Univ* **39**, 207-216 (1964).

12 Umbreit, W. Manometric techniques. (1957).

13 Lowry, O. H., Rosebrough, N. J., Farr, A. L. & Randall, R. J. Protein measurement with the Folin phenol reagent. *Journal of biological chemistry* **193**, 265-275 (1951).
